# Comparative Evaluation of Commercial Alginate Hydrogels: Effects of Viscosity, Polymer Concentration, and Crosslinking on Structural, Mechanical, and Biological Properties

**DOI:** 10.3390/ph19091441

**Published:** 2026-09-11

**Authors:** Azadeh Shahroodi, Valeria Graceffa, Ioannis Manolakis, Patrick Delassus, Liam Morris

**Affiliations:** 1 Department of Mechanical & Industrial Engineering, Atlantic Technological University, Dublin Road, H91 T8NW Galway, Ireland; patrick.delassus@atu.ie (P.D.); liam.morris@atu.ie (L.M.); 2 Department of Medical & Engineering Technologies Centre, Atlantic Technological University, Galway Campus, Dublin Road, H91 T8NW Galway, Ireland; 3 Department of Life Sciences, Atlantic Technological University, Ash Lane, F91 YW50 Sligo, Ireland; valeria.graceffa@atu.ie; 4 Department of Pharmacy & Pharmaceutical Sciences, Atlantic Technological University, Ash Lane, F91 YW50 Sligo, Ireland; ioannis.manolakis@atu.ie

**Keywords:** alginate hydrogel, calcium crosslinking, alginate viscosity grade, dynamic mechanical analysis, pore structure, cell viability

## Abstract

**Background/Objectives:** Alginate hydrogels are widely used in tissue engineering; however, their reported properties vary significantly due to differences in formulations and processing conditions, which limits direct comparison across studies. This study aims to systematically evaluate the relative and combined effects of alginate viscosity grade, polymer concentration, and CaCl_2_ crosslinking concentration on hydrogel structural, mechanical, and biological behaviour. **Methods:** Hydrogels were prepared using three commercially available alginates of low, medium, and high viscosity. Polymer concentration (0.5–2% *w*/*v*) and CaCl_2_ concentration (2.5–10% *w*/*v*) were systematically varied under controlled fabrication conditions. Morphology was analysed using scanning electron microscopy, swelling and water uptake were quantified, mechanical properties were assessed via dynamic mechanical analysis, and cell viability was evaluated using Chinese hamster ovary (CHO) cells encapsulation over 20 days. Statistical analysis was performed using two-way ANOVA. **Results:** Hydrogel properties were governed by non-linear interactions between formulation parameters. CaCl_2_ concentration was identified as the dominant factor influencing structural and biological outcomes, with increasing crosslinking concentration reducing pore size, swelling, and water uptake, and decreasing cell viability by up to ~60%. In contrast, polymer concentration and alginate viscosity grade primarily controlled mechanical behaviour, with increased polymer content and viscosity resulting in higher storage and Young’s moduli. Significant interaction effects confirmed that hydrogel properties are not independently tunable but depend on the combined influence of all parameters. **Conclusions:** Crosslinking concentration dominates structural and biological responses in alginate hydrogels, while polymer parameters modulate mechanical properties within this constraint. These findings establish a formulation-dependent trade-off between mechanical stiffness and cytocompatibility, providing a comparative framework for rational selection of alginate systems based on application-specific requirements.

## 1. Introduction

Tissue-related diseases represent a major challenge in modern medicine, as they can significantly affect patients’ quality of life and may lead to irreversible tissue damage if not adequately treated. Tissue engineering, a branch of regenerative medicine, offers promising approaches for the repair and regeneration of damaged tissues. It combines principles from biotechnology and biomaterials science to restore function using scaffolds, cells, and growth factors [1]. In this context, scaffolds provide a three-dimensional environment that supports cell growth and tissue formation [2].

Different tissues in the body exhibit distinct mechanical and biological characteristics; therefore, scaffold design must be adapted to the specific properties of the target tissue [3,4]. These differences arise from variations in factors such as cellular density, tissue function, and mechanical environment required to support tissue regeneration [5]. For example, the mechanical stiffness of human tissues spans a wide range, from very soft tissues such as brain or fat (~10 Pa) [6,7] to extremely stiff tissues such as cortical bone (~10 GPa) [8,9]. Accordingly, selecting materials that replicate the key structural and functional properties of native tissues is essential to successful tissue engineering strategies.

The influence of scaffold properties on cell behaviour has been widely reported, particularly in relation to mechanical properties (such as storage modulus, loss modulus, and Young’s modulus) [10] and morphological characteristics [11] (such as pore size, porosity, and surface morphology) for both synthetic and natural polymers [5,12]. Among the different scaffold systems investigated, hydrogels have emerged as one of the most widely used materials [13]. Hydrogels are three-dimensional, water-swollen polymer networks that can be synthetic (such as polyacrylamide [PAA], polyvinyl alcohol [PVA], and polyethylene glycol [PEG]) [14] or derived from natural polymers (such as chitosan, alginate, gelatine, and collagen) [2,13]. They are widely used as scaffold materials due to their porous and hydrophilic structure, which facilitates the transport of oxygen and nutrients [15].

Natural and synthetic polymers offer distinct advantages in tissue engineering [12]. Synthetic polymers generally exhibit superior mechanical strength and structural stability; however, they often lack the biocompatibility and bioactivity required to support cell adhesion and proliferation [16]. In contrast, natural polymers are highly regarded for their biodegradability, inherent biocompatibility, and ability to promote cell attachment, making them attractive candidates for diverse applications in tissue engineering [17]. Nevertheless, natural polymers also present limitations, including relatively weak mechanical properties, degradation rates that may not align with tissue regeneration timelines, batch-to-batch variability due to their biological origin [16], and limited control over chemical composition and functional groups. These constraints can hinder their use in load-bearing applications and reduce reproducibility in scaffold fabrication [16].

Among natural polymers, alginate, collagen, and hyaluronic acid are widely investigated natural polymers because of their favorable biocompatibility and suitability for tissue engineering applications [18,19,20]. In addition, alginate is generally considered biocompatible and has been widely investigated for hydrogel-based tissue engineering applications [21] and, when formulated as a hydrogel, enables tunable mechanical behavior by adjusting crosslinking density and composition [22]. Moreover, alginate provides good structural stability and is considerably more cost-effective than many other natural polymers, making it one of the most widely used materials for hydrogel fabrication [23].

The processing conditions for alginate are also relatively mild and straightforward [24] compared with those for polymers such as collagen [19,20] or chitosan [24,25]. For example, chitosan requires acidic conditions for dissolution [25], gelatine depends on temperature-sensitive gelation [26], and collagen often involves complex preparation and neutralisation steps [21]. Altogether, these characteristics make alginate a suitable candidate for producing tunable hydrogels for biomedical applications.

Mechanical testing of alginate hydrogels under tensile [27], compressive [23], and shear conditions [28], as well as under quasi-static [29] and dynamic loading [30,31], has been widely reported. In addition to mechanical characterization, parameters such as porosity [32], pore size, swelling behavior, and water absorption [33] are commonly evaluated to assess scaffold performance, particularly for applications involving cell seeding and growth within hydrogel matrices [32].

The hydrogels under investigation are of particular interest for cartilage and soft tissue engineering applications where a compromise between structural stability, mechanical compliance, and cell viability is required [34,35,36]. The identified formulations may serve as candidate materials for cell encapsulation and 3D tissue models, where control of structural and mechanical properties is important. However, the interpretation and comparison of reported hydrogel properties are complicated by substantial variations in alginate source, formulation parameters, fabrication methods, and testing protocols among studies. As a result, it remains challenging to distinguish the separate contributions of alginate viscosity grade, polymer concentration, and crosslinking conditions on hydrogel performance. Although the effects of alginate composition and crosslinking density have been extensively investigated [37], direct comparative studies of commercially available alginate viscosity grade under identical experimental conditions are still limited [38,39].

Many studies have investigated alginate hydrogels for tissue engineering and bioprinting. Most of these studies have focused on single formulations, specific material properties, or specific biomedical applications. For this reason, it is rare to find direct comparisons of different viscosities of commercial alginate tested under the same conditions. There are also few studies that have investigated how alginate viscosity, polymer concentration, and CaCl_2_ concentration affect structural, mechanical, swelling, and biological properties in a single experiment. Table 1 summarizes key studies and highlights this gap in research.

The novelty of this study lies not in the creation of a new hydrogel formulation, but in the systematic and controlled comparison of three commercially available alginate viscosity grades. In contrast to previous studies that focus on individual alginate systems or isolated formulation variables, this research independently varied the polymer and CaCl_2_ concentrations under identical fabrication and characterization conditions. This methodology allows for the distinction of the relative and combined effects of viscosity, polymer concentration, and crosslinking conditions within a reproducible experimental framework.

This study evaluated the influence of formulation parameters on the morphological, swelling, mechanical, and biological properties of alginate hydrogels. By identifying the parameters that most significantly affect structural stability, mechanical performance, and cell viability, the research establishes a comparative framework for selecting commercially available alginate formulations based on specific tissue-engineering requirements.

## 2. Results

### 2.1. Morphological Analysis

**Figure 1:**
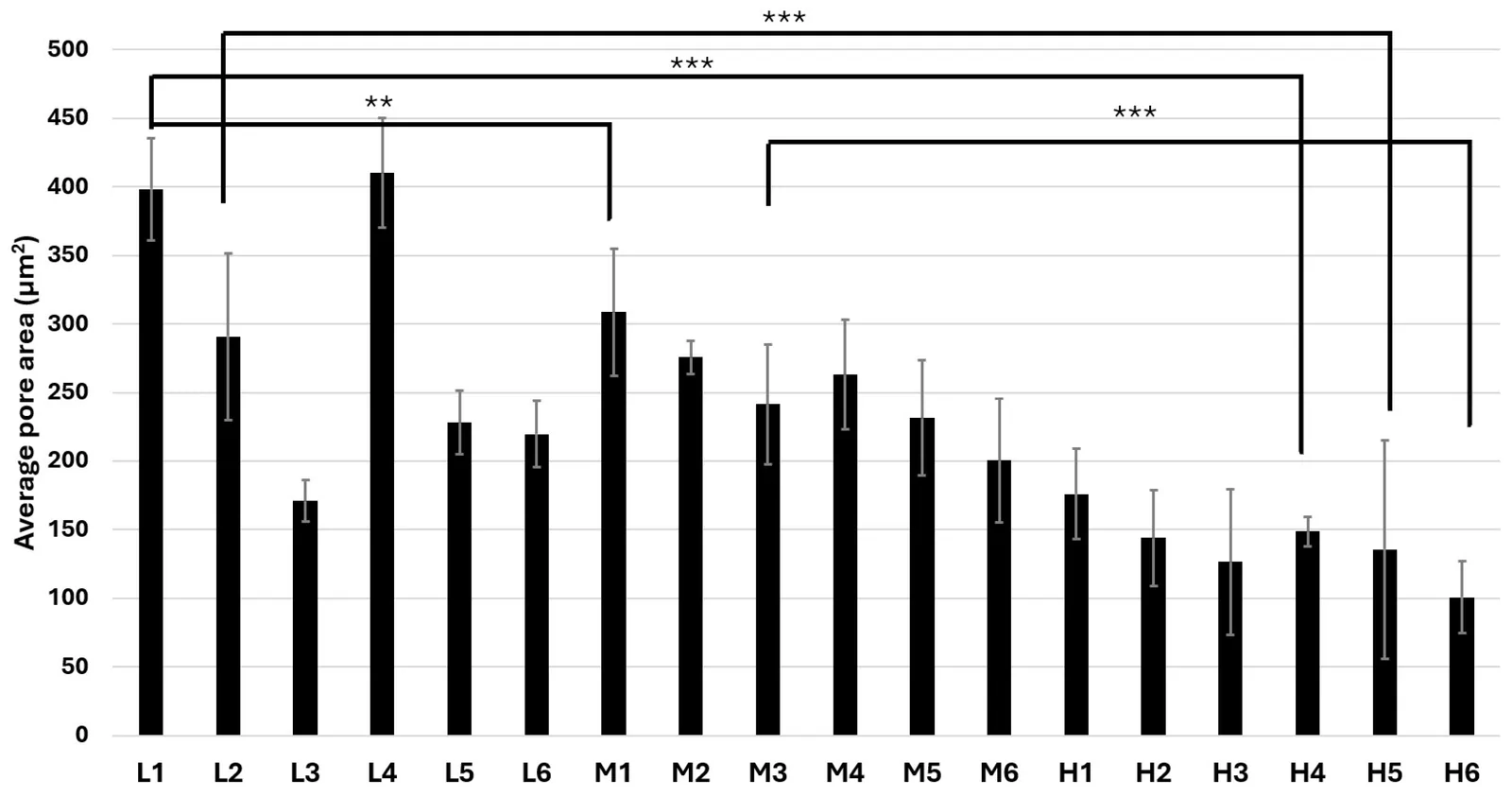
Average pore area of alginate-based hydrogel scaffolds with different alginate viscosity grades, polymer concentrations, and CaCl_2_ concentrations. Data are presented as mean ± SD (*n* = 5). Statistical significance was determined using two-way ANOVA followed by Tukey’s post hoc test (** *p* < 0.01, *** *p* < 0.001).

**Figure 2:**
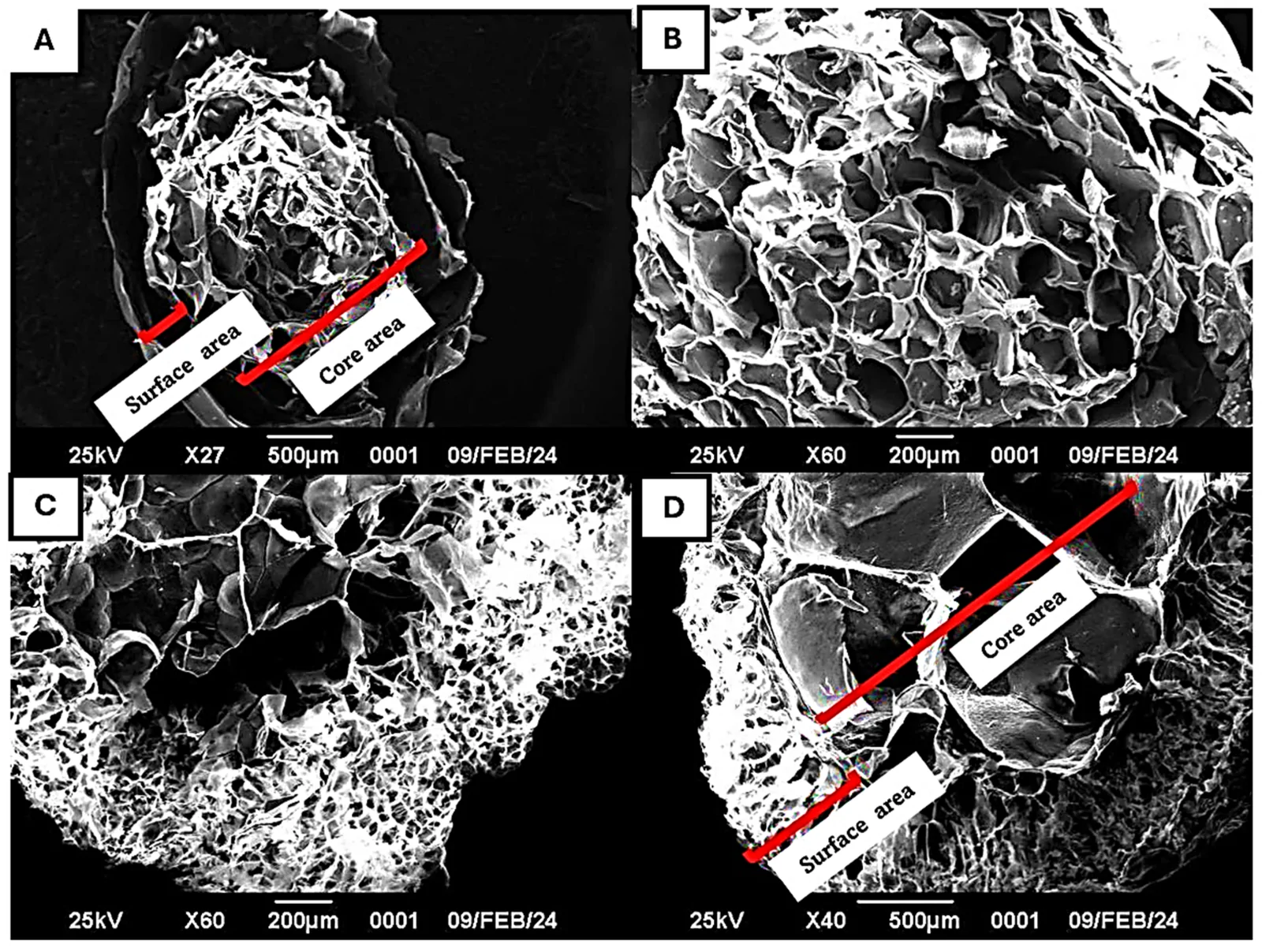
SEM micrographs of freeze-dried alginate-based hydrogel showing differences between surface and core regions. (**A**) Low-viscosity alginate hydrogel, (**B**) Medium-viscosity alginate hydrogel, and (**C**,**D**) High-viscosity alginate hydrogel. Red arrows indicate the surface area and core area of the samples. Differences in pore morphology and density between the outer and inner regions are evident, with the low-viscosity alginate exhibiting more open, interconnected pores than the denser structure of the high-viscosity alginate.

Figure 1 shows that the average pore size generally decreases with increasing CaCl_2_ concentration and alginate viscosity grade. At the typical polymer concentration of 1% (*w*/*v*), two-way ANOVA revealed significant effects of CaCl_2_ concentration (F(2,36) = 26.426, *p* < 0.001) and alginate viscosity grade (F(2,36) = 62.943, *p* < 0.001), accompanied by a significant interaction between these two factors (F(4,36) = 6.556, *p* < 0.001). SEM analysis of freeze-dried samples revealed viscosity-dependent differences in pore organization. Alginate with medium viscosity exhibited a more uniform microstructure, while low- and high-viscosity formulations showed significant core-surface variations (Figure 2). Low-viscosity hydrogels exhibited larger surface pores (30–60 μm) that decreased to 3–4 μm in the interior, whereas high-viscosity hydrogels showed the opposite trend, with smaller surface pores in the core that increased to 40–50 μm. Quantitative pore measurements are summarized in Table 2 (see Table S1). The composition and nomenclature of the hydrogel formulations (L1–H6) are described in Section 4.2.

**Figure 3:**
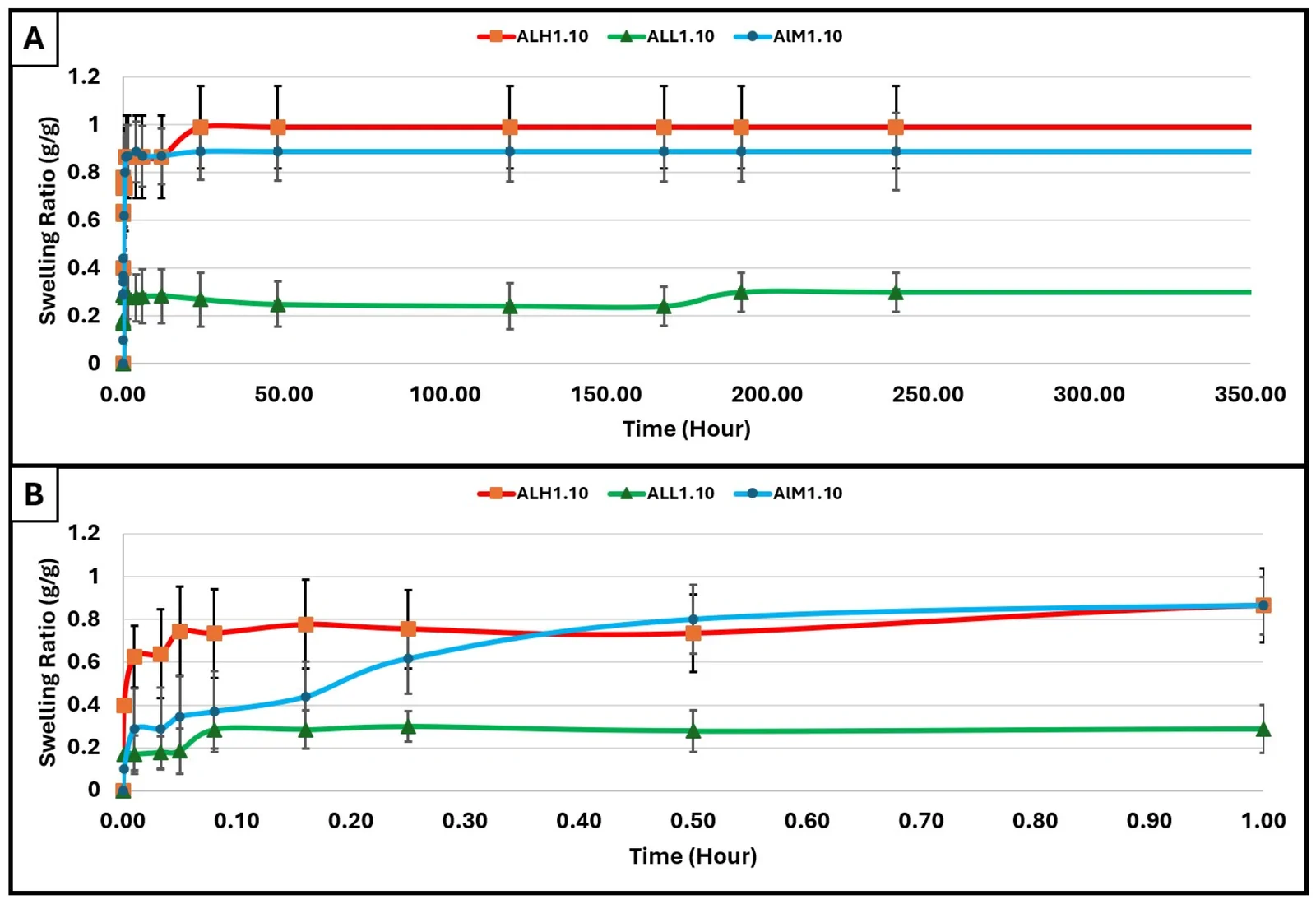
Swelling behaviour of low- (ALL), medium- (ALM), and high-viscosity (ALH) alginate hydrogels at a common polymer concentration of 1% (*w*/*v*) and CaCl_2_ concentration of 10%. (**A**) Long-term swelling behaviour and (**B**) swelling behaviour during the first hour. Data are presented as mean ± SD, (*n* = 5).

The swelling behavior showed a clear dependence on the alginate viscosity grade. To allow a direct comparison between the viscosity grades, low (ALL), medium (ALM) and high (ALH) viscosity alginates were compared at the common polymer concentration of 1% (*w*/*v*) and a CaCl_2_ concentration of 10% (Figure 3). ALH showed a rapid initial swelling, reaching approximately 0.7–0.8 g/g in the first 0.1 h and approaching 1.0 g/g at later times. In contrast, ALM showed a more gradual increase in swelling ratio, reaching approximately 0.9 g/g at later times. ALL showed significantly less swelling over the measurement period, with values remaining at approximately 0.2–0.3 g/g. These results indicate distinct swelling kinetics among the three alginate viscosity grades at the same polymer and crosslinker concentrations.

After transformation, two-way ANOVA at the common polymer concentration of 1% (*w*/*v*) showed significant effects of CaCl_2_ concentration (F(2,36) = 8.856, *p* < 0.001) and alginate viscosity grade (F(2,36) = 12.965, *p* < 0.001) on water uptake. The interaction between CaCl_2_ concentration and viscosity grade was not significant (F(4,36) = 1.901, *p* = 0.132).

**Figure 4:**
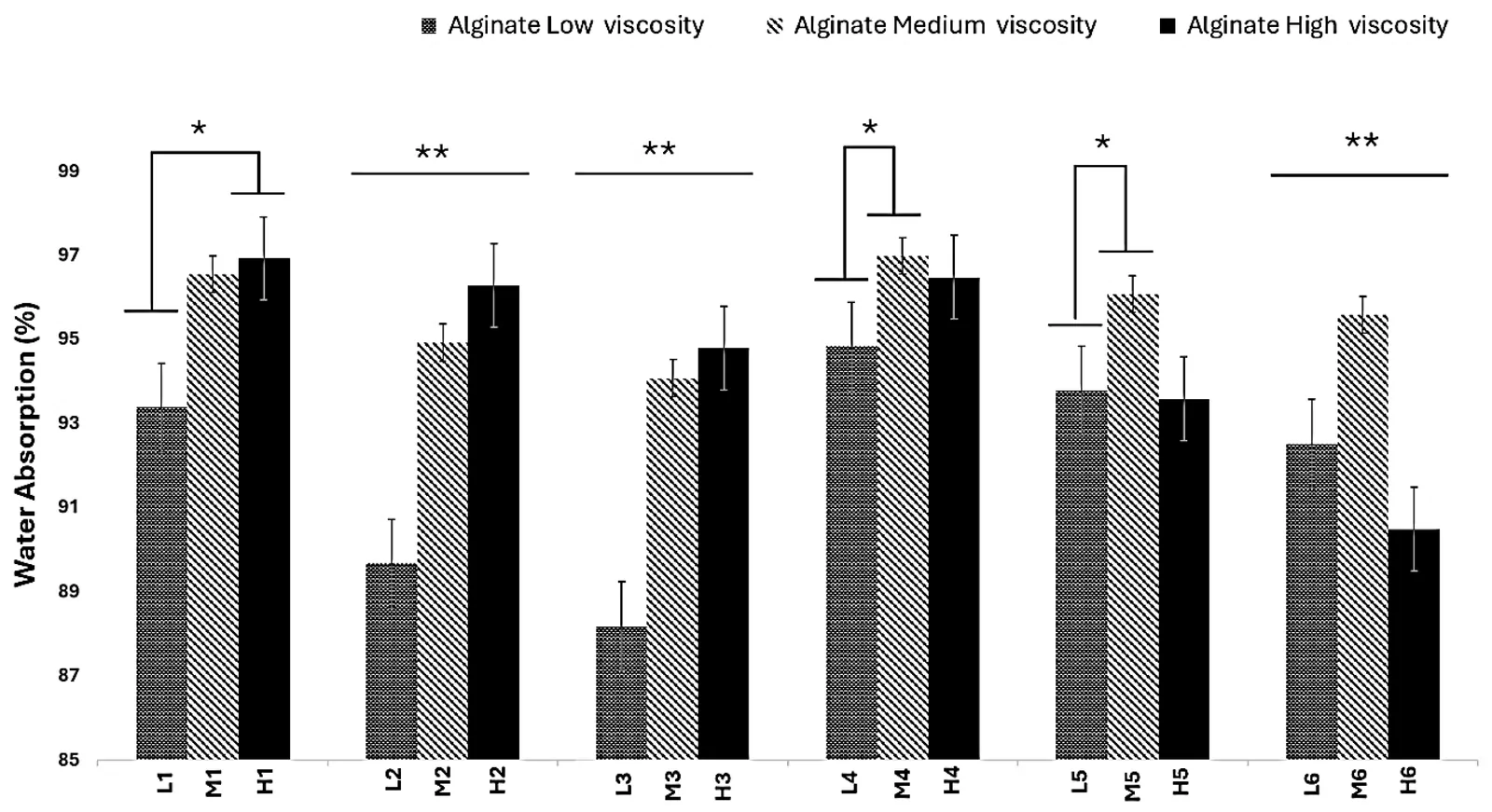
Water absorption (%) of alginate hydrogels prepared with low-, medium-, and high-viscosity alginates at different polymer and CaCl_2_ concentrations. Data are presented as mean ± SD (*n* = 5). Statistical significance was assessed using two-way ANOVA followed by post hoc multiple comparisons, with detailed statistical results provided in Table S2. Asterisks indicate statistically significant differences between the groups compared (* *p* < 0.05, ** *p* < 0.01).

A reduction in water uptake was observed at the highest CaCl_2_ concentrations in the high-viscosity formulations (Figure 4). Separate two-way ANOVAs within each viscosity grade showed that the effects of polymer and CaCl_2_ concentrations on water uptake varied across viscosity grades; detailed statistical results are provided in Table S2.

### 2.2. Mechanical Properties

The storage modulus (E′) varied across formulations, with higher polymer concentrations generally associated with increased E′ values (Table 3). At the common polymer concentration of 1% (*w*/*v*), two-way ANOVA showed significant effects of CaCl_2_ concentration (F(2,36) = 26.487, *p* < 0.001) and alginate viscosity grade (F(2,36) = 184.695, *p* < 0.001), with a significant interaction between the two factors (F(4,36) = 15.657, *p* < 0.001). Separate analyses within each viscosity grade also showed significant effects of polymer concentration on E′ (Table S3).

CaCl_2_ concentration significantly affected storage modulus (E′), although its effect varied with alginate viscosity grade and polymer concentration. Higher CaCl_2_ concentrations were generally associated with increased E′ values. The hysteresis ratio (HR) was also significantly influenced by formulation composition, with the effects of CaCl_2_ and polymer concentration varying among the viscosity grades. (see Table S8).

Loss modulus (E″) was also affected by formulation composition. At the common polymer concentration of 1% (*w*/*v*), two-way ANOVA showed significant effects of CaCl_2_ concentration (F(2,36) = 5.478, *p* = 0.008) and alginate viscosity grade (F(2,36) = 31.648, *p* < 0.001), together with a significant interaction between the two factors (F(4,36) = 3.627, *p* = 0.014). Within the low- and medium-viscosity formulations, polymer concentration significantly affected E″, whereas no significant effect of polymer concentration was detected within the high-viscosity formulations (*p* = 0.883). (See Tables S3 and S4).

**Figure 5:**
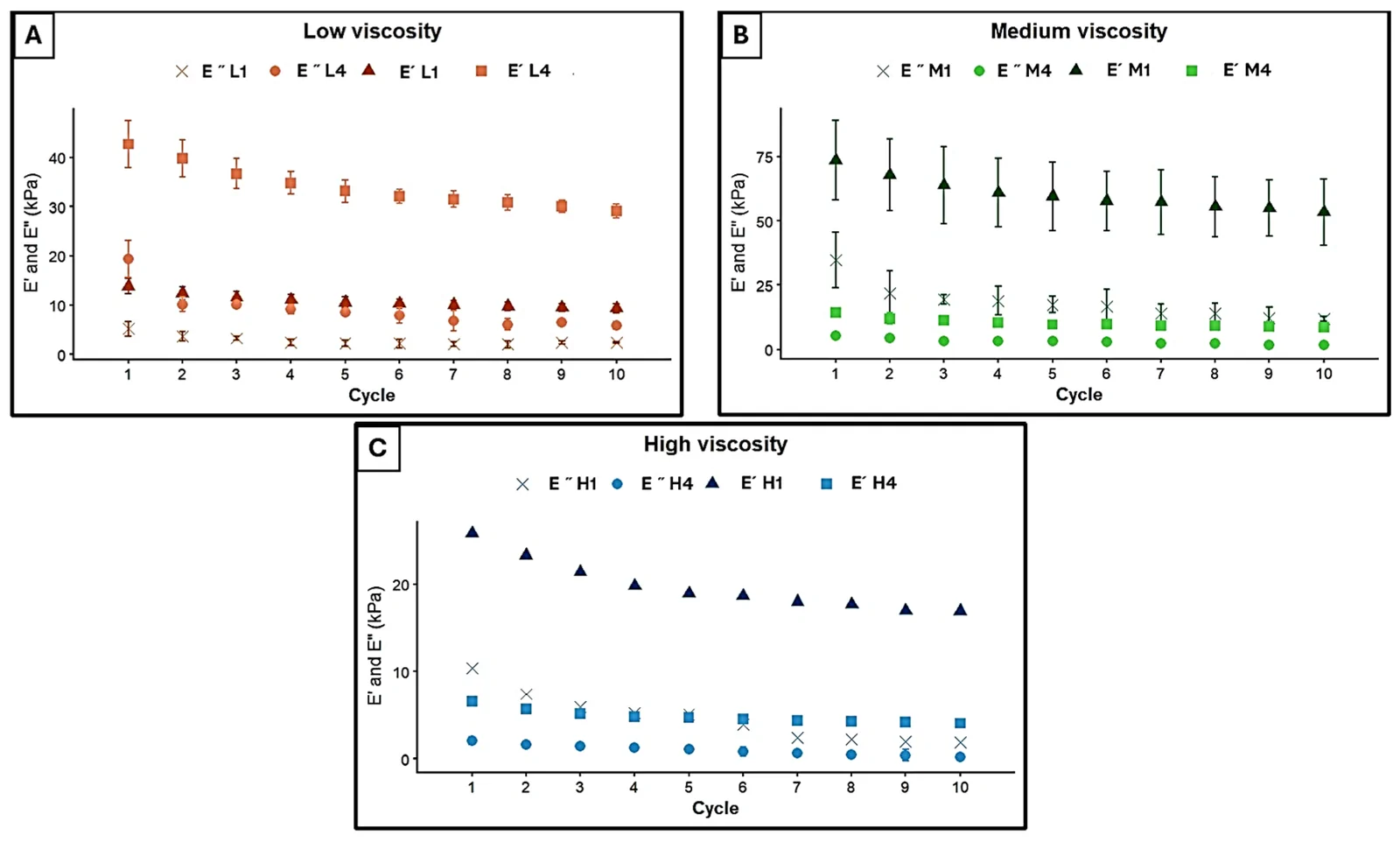
Storage modulus (E′) and loss modulus (E″) over 10 loading cycles for (**A**) low-viscosity, (**B**) medium-viscosity, and (**C**) high-viscosity alginate formulations. Data are presented as mean ± SD (*n* = 5).

**Figure 6:**
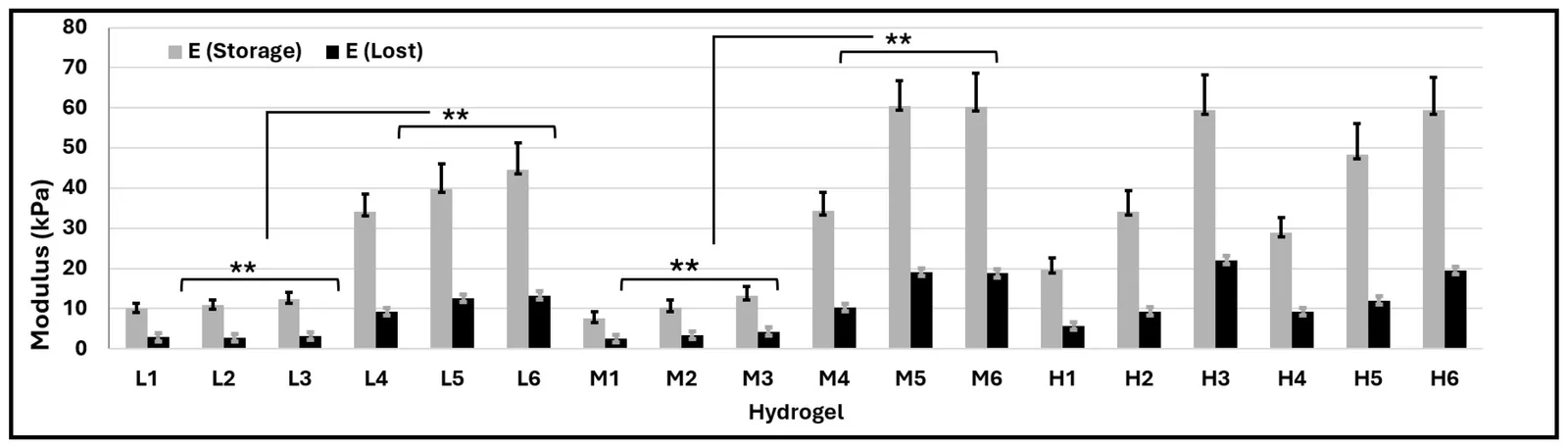
Comparison of storage and loss modulus (E′ and E″) across groups. Double asterisks indicate statistically significant differences ** (*p* < 0.01).

Young’s modulus (YM), obtained from compressive DMA analysis, showed trends consistent with those observed for storage modulus. Because polymer concentration varied across viscosity grades, a direct comparison of the viscosity-grade effect was conducted at the common polymer concentration of 1% (*w*/*v*). At this concentration, two-way ANOVA showed significant effects of CaCl_2_ concentration (F(2,36) = 476.818, *p* < 0.001) and alginate viscosity grade (F(2,36) = 4035.249, *p* < 0.001), together with a significant interaction between the two factors (F(4,36) = 283.291, *p* < 0.001). At 2.5% CaCl_2_, YM values were 14.28 ± 1.97, 15.81 ± 5.31 and 39.91 ± 1.79 kPa for low, medium and high viscosity alginates, respectively. At 5% CaCl_2_ concentration, the corresponding values were 15.47 ± 2.41, 17.30 ± 1.22 and 71.08 ± 9.02 kPa, respectively, while at 10% CaCl_2_ concentration they were 18.15 ± 5.26, 22.94 ± 7.56 and 90.44 ± 11.67 kPa, respectively. These results provide a direct comparison of the three viscosity grades at equivalent polymer concentrations and indicate that under the test conditions, the high-viscosity alginate, especially at higher CaCl_2_ concentrations, exhibits the highest YM. The complete mechanical parameters for all formulations are presented in Table 3 and Figure 5 and Figure 6 (see Table S6).

**Figure 7:**
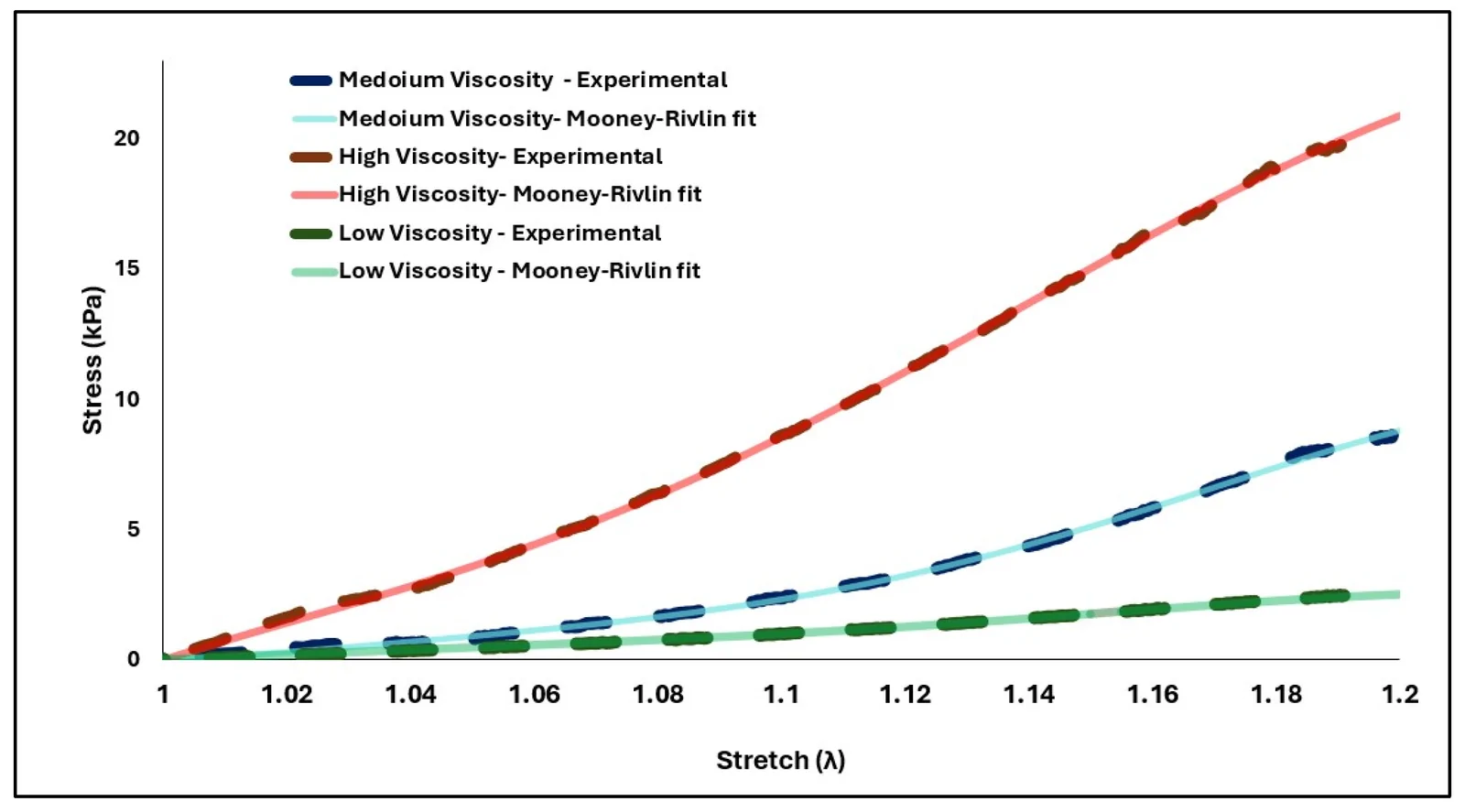
Uniaxial stress–stretch curves of the hydrogel samples. Experimental data (points) are compared with Mooney–Rivlin model fits (dashed lines), demonstrating the nonlinear mechanical response of the materials.

To examine the nonlinear mechanical response of the hydrogels under compression, the experimental stress–stretch data were fitted using the Mooney–Rivlin hyperelastic model. Figure 7 shows the Mooney–Rivlin representation of the compression data together with the fitted curves for hydrogels crosslinked with different CaCl_2_ concentrations. In all samples, stress increased nonlinearly with increasing deformation, consistent with strain-stiffening behaviour commonly reported for ionically crosslinked alginate networks. Hydrogels prepared with higher CaCl_2_ concentrations exhibited steeper stress–stretch responses, reflecting increased resistance to deformation. In the Mooney plot, the data follow approximately linear trends within the analysed deformation range, indicating that the Mooney–Rivlin model provides a good empirical description of the compressive behaviour of these hydrogels.

The fitted Mooney–Rivlin parameters are summarised in Table 4. The coefficients a_10_ and a_01_ are associated with the initial elastic response of the material, whereas the higher-order coefficients (a_20_, a_11_, and a_30_) account for nonlinear contributions to the mechanical behaviour. Although the fitted curves showed a high goodness of fit (R^2^ > 0.999 for all samples), considerable variation in the individual parameter values was observed between formulations, including changes in coefficient sign. This indicates that, while the model accurately captures the overall nonlinear compressive response, the physical interpretation of individual fitting parameters should be treated with caution. Overall, the results suggest that the Mooney–Rivlin model provides a reliable empirical description of the mechanical behaviour, but does not directly reflect the underlying network structure of the alginate hydrogels. The initial modulus (E_0_), calculated from the fitted parameters, showed trends broadly consistent with experimentally measured stiffness values; however, its interpretation is limited to a comparative indicator rather than a direct physical measure.

### 2.3. Cell Viability

**Figure 8:**
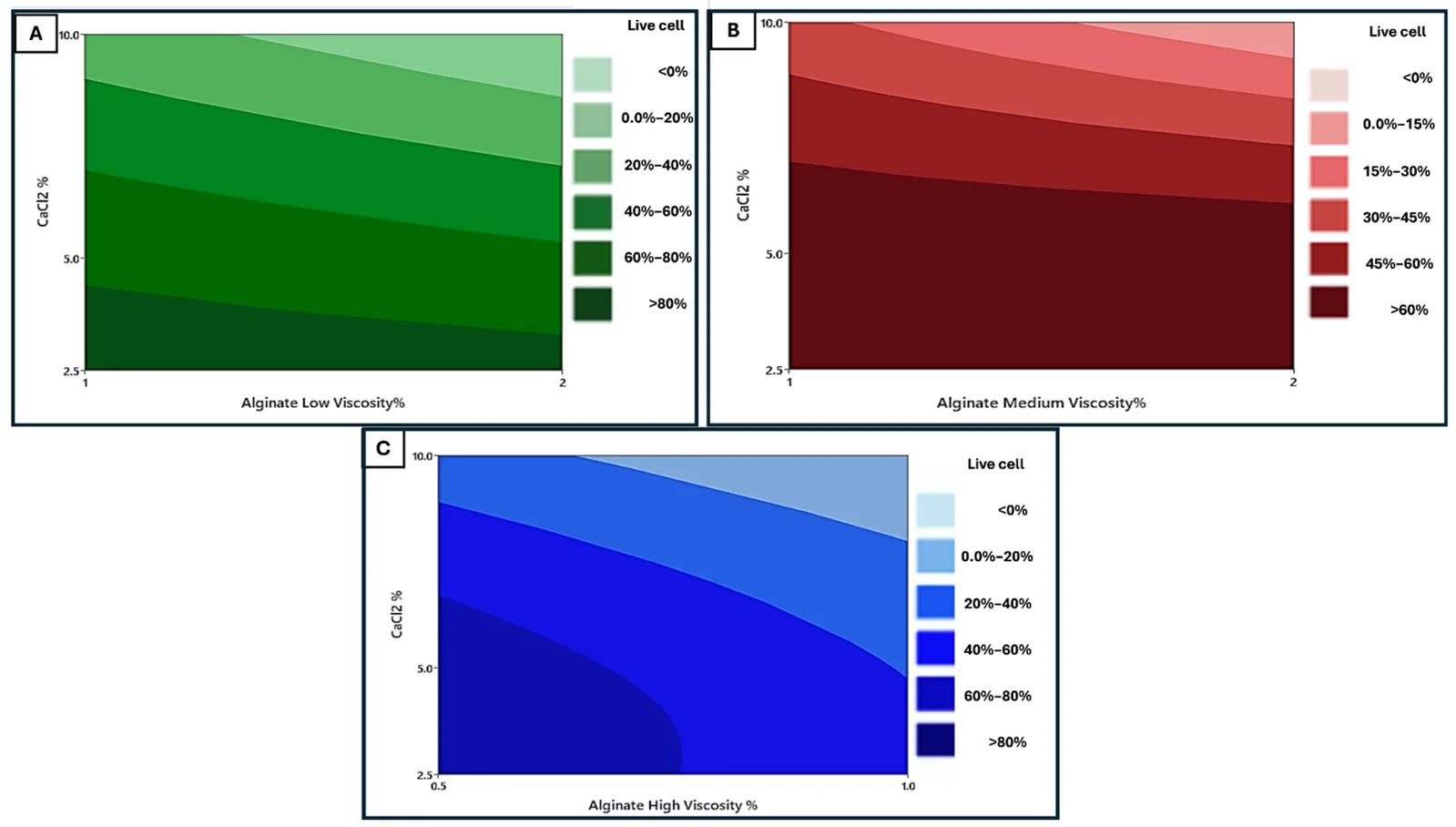
Contour plots showing the percentage of live cells after 20 days of culture as a function of alginate concentration and CaCl_2_ concentration for (**A**) low-viscosity, (**B**) medium-viscosity, and (**C**) high-viscosity alginates. Darker colors indicate higher percentages of live cells, while lighter colors indicate lower cell viability.

**Figure 9:**
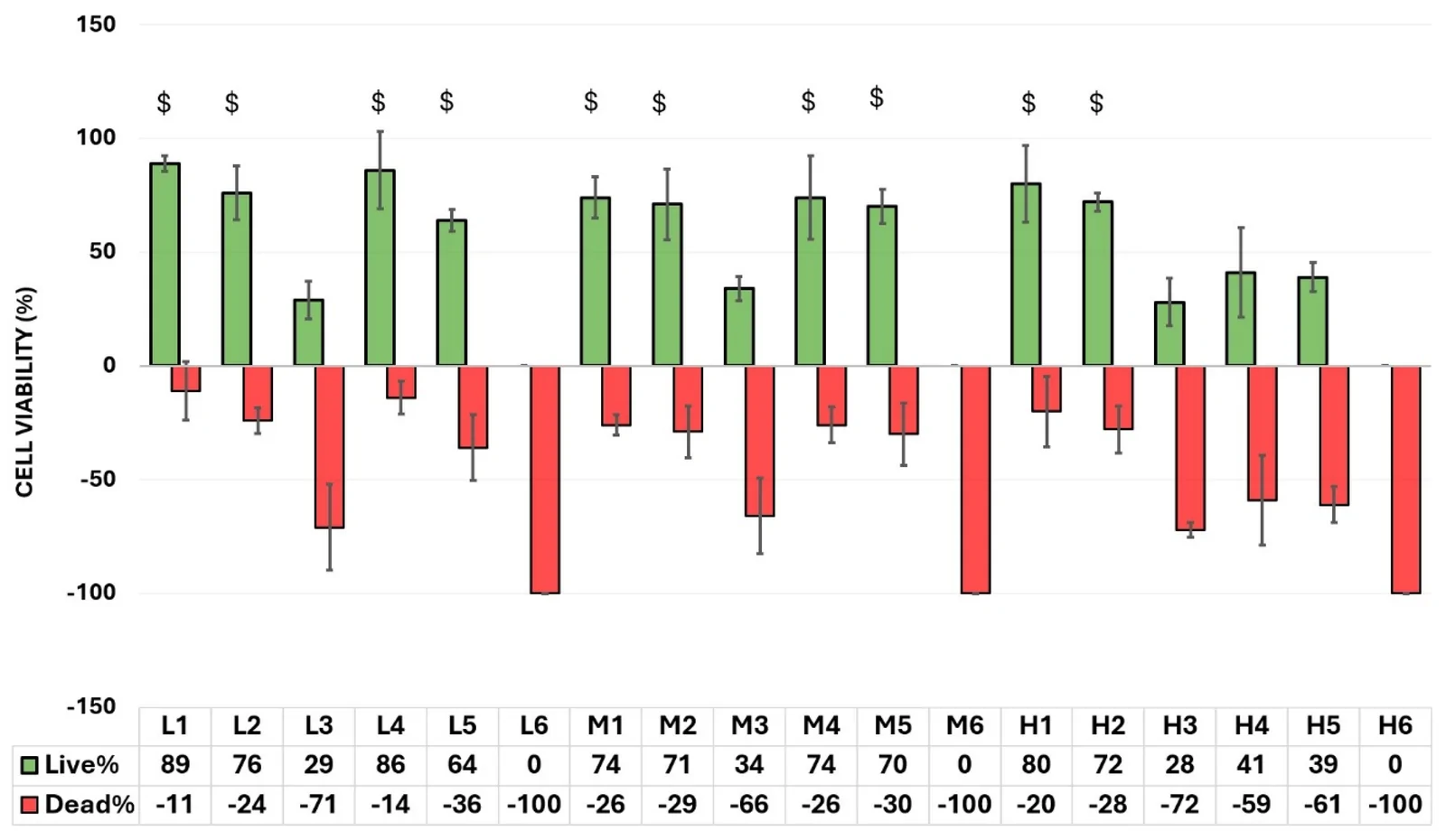
Percentage of live cells (green bars) and dead cells (red bars) after 20 days of culture in alginate hydrogels with different formulations. Data are presented as mean ± SD. Dollar signs ($) indicate formulations in which visible cell colonies were observed after 20 days.

A progressive reduction in the percentage of live cells was observed as CaCl_2_ concentration increased from 2.5% to 10% across all alginate viscosities (Figure 8A–C). Low- and medium-viscosity alginates showed higher viability at lower crosslinker levels, whereas high-viscosity formulations and higher CaCl_2_ concentrations were associated with reduced cell survival. Quantitative analysis (Figure 9) confirmed these trends, with decreasing live cell fractions and increasing dead cell fractions as CaCl_2_ concentration increased. At the common polymer concentration of 1% (*w*/*v*), two-way ANOVA showed significant effects of CaCl_2_ concentration and alginate viscosity grade on the percentage of live cells, together with a significant interaction between these factors (*p* < 0.001).

Separate two-way ANOVAs within each viscosity grade showed significant effects of CaCl_2_ concentration and polymer concentration on cell viability, with significant interactions between these factors. Overall, the magnitude of these effects varied among the three viscosity grades.

Despite these overall effects, formulation-specific differences in cell viability were observed. In general, higher CaCl_2_ concentrations were associated with reduced cell viability across the viscosity grades, while the effect of polymer concentration varied among the different alginate viscosity grades.

Overall, CaCl_2_ concentration had a pronounced influence on cell viability, while alginate viscosity grade and polymer concentration also contributed to the observed differences between formulations (Table 5). These trends are consistent with the observed reduction in pore size at higher crosslinking levels, which may be associated with decreased transport of nutrients and metabolic waste over the 20-day culture period; however, transport properties were not directly measured in this study (see Table S9).

## 3. Discussion

A key contribution of this study is the direct comparison of three commercially available alginate viscosity grades under consistent fabrication and testing conditions. Previous research has often examined individual alginate grades or employed non-standardised crosslinking protocols, making it difficult to distinguish intrinsic material behaviour from methodological variability. By systematically evaluating alginate viscosity grade, polymer concentration, and CaCl_2_ concentration in a controlled experimental framework, the present work demonstrates that hydrogel behaviour is formulation-dependent, with the effects of individual parameters varying across formulations. This comparative framework provides a structured approach to linking formulation variables to mechanical, swelling, and biological performance, enabling more informed optimisation of alginate-based scaffold systems.

Morphological characteristics were strongly influenced by CaCl_2_ concentration, with increasing CaCl_2_ levels associated with reduced pore size. This effect can be explained by the mechanism of alginate gelation, in which Ca^2+^ ions interact with guluronic acid blocks along the polymer chains to form ionic cross-linking regions described by the “egg-box” model. As cross-link density increases, a more compact network structure forms, resulting in smaller pores, consistent with previous reports on Ca^2+^-crosslinked alginate systems [42,43]. This behaviour is commonly attributed to Ca^2+^-mediated network formation, which reduces the available free volume within the hydrogel matrix [40,44,45]. It should be noted, however, that these observations are based on freeze-dried samples and therefore reflect the dried microstructure rather than the native hydrated network. Freeze-drying may alter pore size and morphology by removing water and inducing partial collapse of the hydrogel network. Therefore, SEM images should be interpreted as a comparative assessment of structural differences between formulations rather than as an accurate representation of the hydrated structure. This limitation should also be considered when relating pore morphology to cell behavior and transport-related properties. Despite these limitations, the observed morphological differences provide useful insight into how changes in crosslink concentration affect fluid transport and swelling behavior in the hydrogel network.

Nevertheless, the observed trends indicate that increasing crosslinker concentration leads to more compact structural organisation within the hydrogel matrices. These structural changes help explain the relationship between swelling and water uptake. Swelling was assessed at a fixed CaCl_2_ concentration (10%), where viscosity-dependent differences were most apparent. In contrast, water uptake was more strongly influenced by crosslinker concentration, while alginate viscosity did not show a consistent independent effect across all formulations. The revised analysis showed that the effects of polymer and CaCl_2_ concentrations on water uptake varied among the alginate viscosity grades. This formulation-dependent behaviour indicates that changes in individual formulation variables do not produce uniform responses across all conditions. These interactions indicate that changes in individual formulation variables do not yield proportional or predictable responses across formulations, resulting in nonlinear behavior.

The observed formulation-dependent responses are likely due to alginate viscosity grade, polymer concentration, and CaCl_2_ concentration collectively influencing hydrogel formation. Increasing polymer concentration increases chain density and the number of potential cross-linking sites, while increasing CaCl_2_ concentration increases ionic bond formation. However, the effectiveness of Ca^2^+-mediated cross-linking depends on the local polymer environment [18]. At higher polymer viscosities and concentrations, chain entanglement and reduced molecular mobility may limit the extent to which additional Ca^2^+ ions can alter the network structure [46]. As a result, the effect of increasing CaCl_2_ concentration is inconsistent across formulations, leading to nonlinear, formulation-dependent responses in pore structure, mechanical behavior, water uptake, and cell viability [18,44].

One practical consequence of these formulation-dependent interactions is their influence on the swelling behavior, which plays an important role in scaffold stability. The relatively low swelling ratios observed in the present study may help maintain scaffold stability by limiting excessive hydrogel expansion. Previous studies have similarly reported that increased Ca^2+^-mediated crosslinking and reduced swelling can improve the structural and mechanical stability of alginate hydrogels [40,47].

Taken together, these findings indicate that the influence of viscosity on hydrogel behaviour depends on crosslinking conditions, with its effects becoming less pronounced at higher CaCl_2_ concentrations. Under these conditions, crosslinker concentration is the dominant factor determining structural characteristics. The same trend was observed for mechanical behaviour. Stiffness-related properties, including storage modulus, Young’s modulus, and energy dissipation, generally increased with CaCl_2_ concentration; however, polymer concentration and viscosity also contributed to the observed variability. The revised analyses further showed that the relative effects of CaCl_2_ concentration, polymer concentration, and alginate viscosity grade varied according to the mechanical parameter and formulation conditions.

The non-linear nature of this mechanical behaviour was further examined using hyperelastic constitutive modelling. The Mooney–Rivlin analysis confirmed the non-linear mechanical response of the hydrogels and showed that their compressive behaviour can be described within a hyperelastic framework. However, the fitted parameters did not show consistent physical trends across formulations, indicating that they should be interpreted as empirical fitting parameters rather than direct descriptors of network structure. The fitted coefficients reproduce the observed stress–strain response over the tested deformation range and should not be considered as independent physical constants of the material. Reporting these coefficients supports repeatability and allows for direct implementation of the material model in computational simulations.

Although increased crosslinking generally improved mechanical performance, the resulting changes in network structure also affected the cellular microenvironment. Crosslinking conditions strongly influenced biological outcomes. Higher CaCl_2_ concentrations were associated with reduced cell viability, which may be linked to the observed decrease in pore size and potential limitations in nutrient transport and waste removal. However, transport properties were not measured directly in the present study. In contrast, lower crosslinking conditions supported higher cell viability but exhibited reduced mechanical strength. This demonstrates a clear trade-off between mechanical reinforcement and cytocompatibility, which must be considered in hydrogel design.

Crosslinking concentration had a pronounced influence on cell viability, with higher CaCl_2_ concentrations generally associated with reduced cell survival. Alginate viscosity grade and polymer concentration also contributed to the observed biological response, with their effects varying across formulation conditions. These findings indicate that cell viability is influenced by the combined formulation conditions rather than by a single parameter alone.

The influence of crosslinking concentration on biological response was also reflected in cell morphology. Cells encapsulated within more open and less densely crosslinked hydrogels formed spheroidal aggregates, as illustrated in Section 4.6, consistent with previous observations in three-dimensional culture systems where cell–cell interactions dominate [47]. The presence of these multicellular aggregates suggests that cell behavior is influenced not only by cell–material interactions but also by cell–cell interactions. Consequently, the biological assessment in the present study focused on cell viability rather than proliferation, thereby allowing evaluation of cell survival and cytocompatibility while minimizing variability associated with proliferation dynamics. The interpretation of these biological findings must also be considered in the context of the cellular model used in this study.

CHO cells were chosen because they are an established, robust cell line commonly used for initial evaluation of cell compatibility and assessment of cell survival in biomaterial systems. In the present study, they were used as a primary model to evaluate the overall cell compatibility of hydrogels. However, CHO cells do not fully exhibit cartilage-specific cell behavior. Therefore, future studies should investigate more relevant cell types, such as chondrocytes or mesenchymal stem cells, to further evaluate the potential of these formulations for cartilage tissue engineering applications.

Despite the limitations of using CHO cells, the structural, mechanical, and biological trends identified in the present study remain relevant to cartilage tissue-engineering applications, in which scaffold stiffness, pore structure, and transport behavior may influence chondrogenic activity and cellular responses. The Young’s modulus values of the investigated hydrogels ranged from approximately 14 to 90 kPa, which are lower than those reported for normal mature cartilage tissue but within the ranges typically investigated for soft hydrogel-based cell encapsulation systems. Furthermore, the observed pore sizes may support cell encapsulation while maintaining the structural integrity of the hydrogel network. The results suggest that formulations under moderate crosslinking conditions may provide a more balanced combination of structural stability and biological function for cartilage repair applications. This balance arises from the interdependent effects of formulation parameters on hydrogel structure, mechanics, and biological performance.

Overall, the results demonstrate that hydrogel properties are not independently tunable. Instead, structural, mechanical, and biological outcomes depend on the formulation conditions, with CaCl_2_ concentration, polymer concentration, and alginate viscosity grade contributing to different extents across the measured properties. This establishes a formulation-dependent trade-off between stiffness and cell viability and highlights the importance of considering parameter interactions when designing alginate hydrogels.

These findings provide practical guidance for selecting alginate formulations that balance structural stability, mechanical performance, and biological response. The comparative framework enables the identification of formulations with balanced structural, mechanical, and biological properties that can be tailored to different tissue-engineering requirements.

Among the formulations evaluated, medium-viscosity alginate combined with moderate CaCl_2_ concentration provided a more balanced performance across structural, mechanical, and biological metrics, suggesting its suitability for applications requiring mechanical compliance alongside adequate cell viability.

While these findings highlight promising formulation strategies, several limitations should be acknowledged. Structural analysis was based on freeze-dried samples, which may not fully represent the hydrated network. The formulation space was restricted to a defined range of polymer and crosslinker concentrations. Furthermore, commercially defined viscosity grades can encompass broad viscosity ranges; therefore, the behaviour observed for the specific alginate grades evaluated in this study may not represent all materials within the same viscosity category, and application-specific selection will require experimental optimisation. Biological assessment was limited to cell viability without functional assays. Future studies incorporating direct transport measurements, broader compositional ranges, and lineage-specific functional assessments would further strengthen the applicability of these findings.

## 4. Materials and Methods

### 4.1. Materials

Sodium alginate powders of three viscosity grades—low (<240 mPa·s), medium (240–3500 mPa·s), and high (>3500 mPa·s)—were sourced from Thermo Fisher Scientific (Currabinny, Carrigaline, Co. Cork, Ireland; CAS No. 9005-38-3). The alginates were classified according to the manufacturer’s viscosity grades. Molecular weight distribution data (Mw, Mn, and PDI) were not provided by the supplier. Cell culture reagents, including Dulbecco’s Modified Eagle Medium (DMEM, F-12 HEPES; Product No. 11330057), fetal bovine serum (FBS; Product No. 10270106), trypsin-EDTA solution (Product No. 12604013), and penicillin-streptomycin solution (Product No. 15140122), were obtained from Gibco (Life Technologies Limited, Paisley, UK).

### 4.2. Fabrication of Hydrogels

Sodium alginate was dissolved in deionized water at the required concentrations by gradually adding the polymer powder under continuous magnetic stirring (200 rpm) for 30 min at room temperature (RT). The solutions were then stirred for 2–3 h to ensure complete dissolution. Calcium chloride (CaCl_2_) solutions were prepared by dissolving the appropriate amount of salt in deionized water at the specified concentrations. Hydrogels were formed by dispensing the alginate solution into a CaCl_2_ crosslinking bath using a custom-fabricated chamber. The constructs were maintained in the CaCl_2_ solution for 10–15 min at room temperature to allow ionic gelation and were subsequently transferred to sterile Falcon tubes for further experiments.

Higher concentrations of high-viscosity alginate were not investigated due to difficulties in preparing homogeneous solutions. The selected CaCl_2_ concentrations were used to generate distinct crosslinking conditions across formulations. The hydrogel formulations are summarised in Table 6.

### 4.3. SEM Observation

The morphology of the hydrogels was examined using a JEOL JSM-6490LV scanning electron microscope (JEOL Ltd., Tokyo, Japan). Following gelation and crosslinking, the scaffolds were frozen at −80 °C for 24 h and then freeze-dried under vacuum (10 Pa) for 48 h. Dried samples were sectioned with a sharp blade and mounted onto SEM stubs. Imaging was performed at an accelerating voltage of 20–25 kV. High-resolution micrographs were obtained to assess surface morphology and microscale structural features of the hydrogels. Pore diameter and area were quantified using ImageJ software (version 1.54; U.S. National Institutes of Health, Bethesda, MD, USA). It should be noted that freeze-drying alters the native hydrated structure of hydrogels; therefore, the observed pore architecture reflects the dried state rather than the in-situ microstructure. For each formulation, five independent hydrogel specimens (*n* = 5) were analysed by SEM, with pore measurements obtained from each specimen.

### 4.4. Water Uptake and Swelling Measurement

The water uptake and swelling capacity of the porous scaffolds were evaluated by immersing the samples in phosphate-buffered saline (PBS, pH 7.4). For each formulation, five independent hydrogel specimens (*n* = 5) were evaluated for water uptake and swelling measurements.

For water uptake measurements, hydrogels were immersed in PBS at room temperature and weighed periodically until two consecutive measurements showed no further change in swollen weight. The equilibrium swollen weight was recorded as W_s_. The scaffolds were then dried in an oven at 40 °C for 24–48 h, and the dry weight (W_d_) was measured [48]. Water uptake was calculated as:

(1) $$\mathrm{Water}\;\mathrm{uptake}=\frac{W_{s}-W_{d}}{W_{d}}$$

For swelling ratio measurements, dried hydrogel samples were immersed in deionized water, and their swollen weight was recorded at specific time intervals (1 min to 7 days). At each time point, samples were gently blotted to remove surface moisture before weighing. The swelling ratio was calculated as:

(2) $$\mathrm{Swelling}\;\mathrm{ratio}=\frac{W_{s}}{W_{d}}$$

### 4.5. Dynamic Mechanical Analysis (DMA)

The dynamic mechanical properties of the alginate composite hydrogels were evaluated using a DMA 3200 instrument (TA Instruments, ElectroForce Systems Group, Eden Prairie, MN, USA), in unconfined compression mode at room temperature. Cylindrical samples (8 mm diameter, 2–4 mm height) were fully swollen in deionized water before testing, with thickness measured beforehand and hydration maintained throughout the procedure. Due to the low stiffness of the hydrogels, sample diameters were measured using a Keyence VHX-700 digital microscope (KEYENCE UK Ltd., Milton Keynes, UK), to minimise measurement error. Five independently prepared specimens from each formulation were subjected to DMA testing (*n* = 5).

A compressive strain of 20% was applied at a frequency of 1 Hz for 10 cycles, based on previous studies [45] that have used similar conditions to assess hydrogel behaviour under dynamic loading. Storage modulus (*E*′), loss modulus (*E*″), and tangent delta (tan δ) were calculated from the DMA stress–strain curves [49]. Each hydrogel formulation was tested in five replicates to ensure reproducibility and statistical reliability.

For each loading cycle, the maximum strain (*ɛ_max_*) and corresponding maximum stress (*σ_max_*) were determined. The time difference between these peak values was used to calculate the phase lag (*ΔT*) between the stress and strain signals. The storage modulus (*E*′) and loss modulus (*E*″) were then calculated using the following equations:

(3) $$E^{\prime }=\frac{\sigma_{\max}\cos\left(\omega_{\Delta T}\right)}{\varepsilon_{max}\;}\;$$

(4) $$E^{\prime \prime }=\frac{\sigma_{max}sin(\omega_{\Delta T})}{\varepsilon_{max}}$$

where the angular frequency (*ω*) was determined using the equation:

(5) ω=2πf

The complex modulus (E*) was calculated based on the storage and loss module using the following equation:

(6) $$E^{*}=\sqrt{{\left(E^{\prime }\right)}^{2}+{\left(E^{\prime \prime }\right)}^{2}}$$

Finally, the phase angle (*δ*) was obtained from:

(7) δ=ωΔT

For each cycle, the hysteresis energy (*H*) was calculated as the absolute difference between the work done during the loading (*A_L_*) and unloading (*A_U_*) phases, using the following equation [50]:

*H* = *A_L_* − *A_U_* (8)

The hysteresis ratio (*HR*) was then calculated as:

(9) $$HR=\frac{H}{A_{L}}$$

Nonlinear compressive behavior was observed in the strain range of interest (≤20%). Therefore, the five-parameter Mooney–Rivlin model was chosen to describe the mechanical response of the hydrogels, as this formulation provides additional flexibility for representing nonlinear hyperplastic behaviour through higher-order terms in the strain invariants [51], while providing a more flexible representation than simpler models such as the neo-Hookean model [52]. At the same time, it avoids the additional complexity and potential overfitting associated with higher-order models such as the Ogden model [53], which are typically applied over larger deformation ranges. The nonlinear compressive behavior of the hydrogels was subsequently analyzed using the Mooney–Rivlin strain energy density function (*W*), given by the following expression [51,54]:

(10) $$W=\sum_{i,j}^{\alpha }a_{ij}\;{\left(I_{1}-3\right)}^{i}{\left(I_{2}-3\right)}^{j}$$

For the five-parameter formulation, this expression expands to [55]:

(11) $$W=a_{10\;}\left(I_{1}-3\right)+a_{01\;}\left(I_{2}-3\right)+a_{11\;}\left(I_{1}-3\right)\left(I_{2}-3\right)+a_{20\;}{\left(I_{1}-3\right)}^{2}+a_{30\;}{\left(I_{1}-3\right)}^{3}$$

where a_10_, a_01_, a_11_, a_20_, and a_30_ are material coefficients obtained through fitting of the experimental stress–stretch data. The initial modulus (*E*_0_) was calculated from the Mooney–Rivlin parameters as [51]:

(12) $$E_{0}=6(a_{10}+a_{01})$$

which represents the small-strain elastic response of the hydrogel.

For uniaxial data for the principal stretch direction $\lambda_{1}$, the first invariant *I*_1_ is.

(13) $$I_{1}=\lambda_{1}^{2}+\frac{2}{\lambda_{1}}$$

And the second invariant *I*_2_ is.

(14) $$I_{2}=2\lambda_{1}+\frac{1}{\lambda_{1}^{2}}$$

### 4.6. In Vitro Cytotoxicity and Cell Culture

Chinese hamster ovary (CHO) cells were cultured under standard conditions at 37 °C in a humidified atmosphere containing 5% CO_2_, with the medium refreshed every 2–3 days. CHO cells were used as a model cell line to assess cytocompatibility within the hydrogel matrices. Cells were maintained in Dulbecco’s Modified Eagle Medium (DMEM) supplement, 10% (*v*/*v*) fetal bovine serum (FBS), 0.06% (*w*/*v*) penicillin, and 0.1% (*w*/*v*) streptomycin. Once cells reached at least 70% confluency, they were detached using 0.05% (*w*/*v*) trypsin and 0.02% (*w*/*v*) EDTA, then resuspended in fresh medium for further procedures. All cell culture reagents were obtained from Gibco™.

Alginate and CaCl_2_ solutions, deionized (DI) water, and molds were autoclaved to ensure sterility. Sterilization was performed in an autoclave chamber at 1–1.2 bar and 121–124 °C for 15 min.

**Figure 10:**
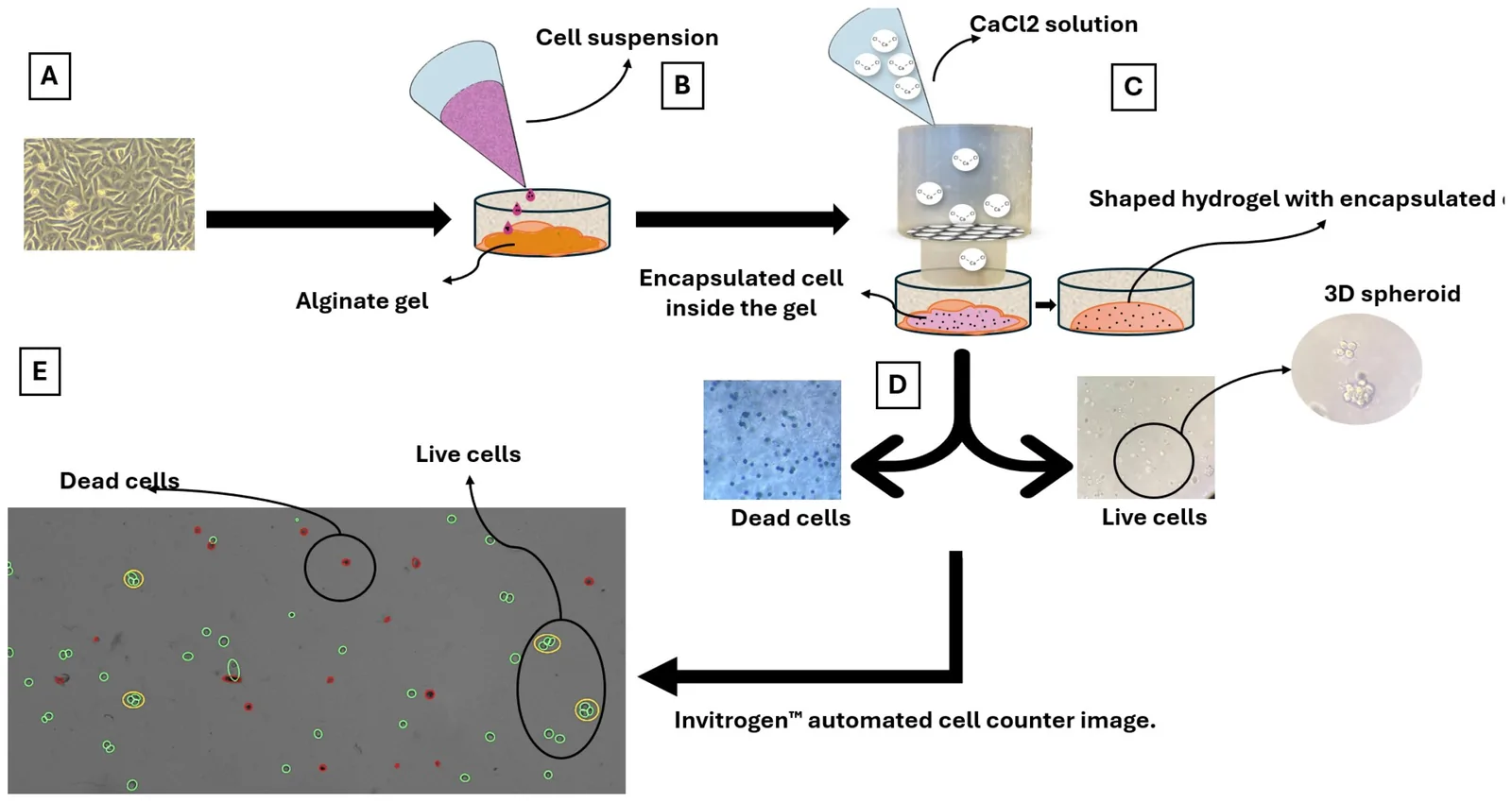
Schematic representation of the process of encapsulating cells in alginate hydrogels and subsequent cell viability analysis. (**A**) Cells were collected, counted, and prepared for encapsulation at a confluence of approximately 70%. (**B**) The prepared cell suspension was gently mixed with the alginate hydrogel precursor solution to ensure uniform cell distribution. (**C**) The alginate mixture containing cells was crosslinked with a CaCl_2_ solution to form a stable hydrogel network. The hydrogels were then placed in a well plate for incubation and further analysis. (**D**) After encapsulation, live and dead cells were counted using a Countess™ automated cell counter. Representative microscopic images of live and dead cells. Live cells formed 3D spheroids in the hydrogel matrix. (**E**) Representative live/dead cell staining images of encapsulated cells, where live cells are green and dead cells are red, indicating compromised membrane integrity.

CHO cells used for encapsulation were collected during the logarithmic growth phase after the third passage. The encapsulation procedure, illustrated in Figure 10, involved pipetting alginate hydrogel into the bottom section of the mold, mixing it with the cell suspension, and sealing it with the top mold piece. CaCl_2_ solution was then added to initiate crosslinking, and the setup was left undisturbed for 5 min. The formed scaffolds were subsequently transferred to culture wells.

Cell viability and colony formation within the scaffolds were assessed on days 1, 3, 5, 7, 10, 12, 15, and 20. Live and dead cells were quantified using the countess™ 3 Automated Cell Counter (Invitrogen™, Thermo Fisher Scientific, Waltham, MA, USA). Formulations that did not support cell survival beyond 48 h were excluded due to lack of measurable viability endpoints. Five separate scaffolds were analysed for each formulation (*n* = 5).

Notably, the highest concentration tested for each alginate viscosity grade was found unsuitable for cell viability and was therefore removed from the experimental dataset.

### 4.7. Statistical Analyses

Statistical analyses were performed using IBM SPSS Statistics version 28 (IBM Corp., Armonk, NY, USA). Normality of the data was assessed using the Shapiro–Wilk and Kolmogorov–Smirnov tests. The water absorption and dynamic mechanical analysis (DMA) data sets deviated from normality and were therefore logarithmically or square-root-transformed before analysis, while the remaining data sets met the assumption of normality without transformation.

Since the polymer concentration levels were not the same across the three alginate viscosity grades, the experimental design was not considered a full three-factor factorial design. Comparisons among low-, medium-, and high-viscosity alginates at the common polymer concentration of 1% (*w*/*v*) were performed using two-way analysis of variance, with alginate viscosity grade and CaCl_2_ concentration as factors. The effects of polymer concentration were assessed separately at each viscosity grade using two-way ANOVA, with polymer concentration and CaCl_2_ concentration as factors. As a result, the three-way interaction of alginate viscosity grade × polymer concentration × CaCl_2_ was not estimated. In cases where significant effects were identified, the Tukey HSD post hoc test was used for multiple comparisons. Statistical significance was defined as *p* < 0.05. Each formulation contained five observations (*n* = 5), with the nature of the replicates specified in the relevant sections of the experimental methods.

## 5. Conclusions

CaCl_2_ concentration exerted a strong influence on pore architecture and cell viability in alginate hydrogels, with average pore area decreasing from approximately 410 to 101 µm^2^ and cell viability decreasing from 89% to complete loss of viable cells under the most highly crosslinked conditions. In contrast, alginate concentration had a greater effect on mechanical behaviour, with Young’s modulus ranging from approximately 14 to 94 kPa, while viscosity provided an additional modulatory contribution.

The formulation parameters acted synergistically, demonstrating that hydrogel properties could be tuned through coordinated changes in polymer composition and crosslinking conditions to achieve different structural, mechanical, and biological responses. This provides a comparative basis for selecting alginate formulations where a balance between structural characteristics and mechanical performance is required. The proposed comparative framework may support the rational development and optimisation of alginate-based scaffold systems for tissue engineering applications.

## Figures and Tables

**Table 1:** Scope of representative alginate hydrogel studies compared with the present investigation.

Study	Scope and Remaining Gap
Gorroñogoitia et al. (2022) [35]	The study primarily examined the effects of alginate viscosity grade and concentration on rheology, printability, and compression. In contrast, CaCl_2_ concentration and biological performance were not systematically investigated.
Malektaj et al. (2023) [40]	Various crosslinking cations were evaluated through equilibrium swelling and dynamic mechanical analysis (DMA), with alginate concentration and viscosity grade held constant.
Li et al. (2023) [36]	The multicomponent GelMA/alginate/PEGDMA/xanthan gum bioink was assessed for printability, compressive properties, degradation, and cytocompatibility, rather than for direct comparison with commercial alginate grades.
Savić Gajić et al. (2023) [41]	The concentrations of alginate and CaCl_2_ were changed to improve water retention and swelling. However, the study did not examine mechanical or biological properties or test different alginate viscosity grades.
Present study	We systematically compared commercial alginate viscosity grades by varying polymer and CaCl_2_ concentrations separately. This approach allowed us to assess structure, properties, and biological effects under the same preparation and testing conditions.

**Table 2:** Quantitative analysis of pore properties of alginate-based hydrogels with different CaCl2 compositions and concentrations. Pore area is expressed as mean ± SD (µm^2^). ImageJ (version 1.54) was used to measure the maximum and minimum pore axis/diameter from SEM images.

Hydrogel	Average Pore Area (± SD) (µm^2^)	Maximum Pore Diameter/Axis (µm)	Minimum Pore Diameter/Axis (µm)
L1	398.45 ± 37.25	61.54	4.77
L2	290.70 ± 61.05	45.89	4.55
L3	171.06 ± 15.34	38.79	3.30
L4	410.23 ± 39.95	52.63	5.41
L5	228.20 ± 22.93	38.79	3.58
L6	219.77 ± 24.09	65.14	5.30
M1	308.68 ± 46.27	47.89	4.87
M2	275.69 ± 12.31	46.00	4.54
M3	241.50 ± 43.57	44.28	4.22
M4	263.10 ± 39.83	41.38	4.59
M5	231.51 ± 41.84	40.54	4.38
M6	200.42 ± 44.96	39.77	4.20
H1	176.02 ± 32.77	44.04	3.69
H2	143.94 ± 34.87	38.99	3.68
H3	126.58 ± 53.26	41.10	3.51
H4	148.74 ± 10.88	48.25	3.64
H5	135.85 ± 79.84	50.02	4.31
H6	100.74 ± 26.05	34.65	3.67

**Table 3:** DMA results for alginate hydrogels with varying alginate viscosity grade, polymer concentration, and CaCl_2_ concentration. Parameters are presented as mean ± SD.

Hydrogel	E′ (Storage Modulus) (kPa)	E″ (Loss Modulus) (kPa)	E* (Complex Modulus) (kPa)	Phase Angle (δ)	Hysteresis Ratio (HR)	Hysteresis kJ/m^3^	Young’s Modulus (kPa) 20% Compression Strain
L1	10.00 ± 1.28	2.88 ± 0.92	10.44 ± 1.48	0.27 ± 0.05	0.74 ± 0.02	0.09 ± 0.03	14.28 ± 1.97
L2	10.81 ± 1.43	2.76 ± 0.99	11.19 ± 1.63	0.24 ± 0.04	0.68 ± 0.01	0.08 ± 0.03	15.47 ± 2.41
L3	12.37 ± 1.63	3.15 ± 1.12	12.79 ± 1.86	0.24 ± 0.04	0.72 ± 0.01	0.11 ± 0.03	18.15 ± 5.26
L4	34.07 ± 4.44	9.25 ± 3.85	35.40 ± 5.30	0.25 ± 0.06	0.71 ± 0.01	0.29 ± 0.11	52.84 ± 6.79
L5	39.88 ± 6.17	12.55 ± 4.59	41.91 ± 7.26	0.29 ± 0.04	0.80 ± 0.01	0.39 ± 0.19	67.89 ± 12.66
L6	44.49 ± 6.73	13.29 ± 5.40	46.56 ± 7.86	0.28 ± 0.07	0.67 ± 0.02	0.35 ± 0.17	75.62 ± 4.53
M1	7.49 ± 1.66	2.52 ± 0.97	7.93 ± 1.87	0.31 ± 0.05	0.93 ± 0.02	0.07 ± 0.04	15.81 ± 5.31
M2	10.32 ± 1.74	3.41 ± 0.89	10.89 ± 1.91	0.28 ± 0.05	0.87 ± 0.03	0.11 ± 0.05	17.30 ± 1.22
M3	13.20 ± 2.21	4.32 ± 1.76	13.93 ± 2.64	0.31 ± 0.06	0.89 ± 0.01	0.14 ± 0.07	22.94 ± 7.56
M4	34.31 ± 4.69	10.32 ± 3.59	35.94 ± 5.45	0.29 ± 0.06	0.77 ± 0.01	0.29 ± 0.13	50.12 ± 3.11
M5	60.44 ± 6.35	19.10 ± 5.92	63.51 ± 7.88	0.30 ± 0.05	0.76 ± 0.01	0.65 ± 0.23	93.69 ± 11.66
M6	60.24 ± 8.43	18.80 ± 7.30	63.3 ± 10.38	0.29 ± 0.05	0.83 ± 0.01	0.57 ± 0.29	82.15 ± 1.62
H1	19.78 ± 2.93	5.65 ± 2.01	20.61 ± 3.37	0.27 ± 0.05	0.92 ± 0.04	0.17 ± 0.07	21.35 ± 3.84
H2	34.22 ± 5.04	9.29 ± 3.48	35.52 ± 5.75	0.25 ± 0.05	0.78 ± 0.01	0.27 ± 0.12	43.75 ± 13.44
H3	59.40 ± 8.76	22.05 ± 7.88	61.23 ± 10.74	0.32 ± 0.06	0.87 ± 0.02	0.81 ± 0.33	65.01 ± 7.34
H4	28.95 ± 3.67	9.20 ± 2.95	30.51 ± 4.33	0.30 ± 0.06	0.83 ± 0.02	0.03 ± 0.01	39.91 ± 1.79
H5	48.31 ± 7.73	12.05 ± 4.03	49.91 ± 8.37	0.24 ± 0.04	0.80 ± 0.04	0.05 ± 0.02	71.08 ± 9.02
H6	59.33 ± 8.24	19.47 ± 10.62	61.67 ± 10.27	0.27 ± 0.12	0.74 ± 0.01	0.05 ± 0.02	90.44 ± 11.67

**Table 4:** Mooney–Rivlin model parameters (a10, a01, a20, a11, a30) obtained from fitting the uniaxial stress–stretch data of the samples. Values are presented as mean ± fitting error. The sum (a10 + a01) represents the initial shear modulus, and R^2^ indicates the goodness of fit.

	a_10_ (kPa)	a_01_ (kPa)	a_20_ (kPa)	a_11_ (kPa)	a_30_ (kPa)	E_0_ = 6 (a_01_ + a_10_)	R^2^
L1	11.61 ± 1.82	−10.20 ± 1.91	293.77 ± 103.58	−316.95 ± 116.03	−136.99 ± 41.06	8.50	0.99
L2	11.99 ± 4.76	−11.42 ± 6.65	125.90 ± 9.77	−141.92 ± 8.68	−43.71 ± 33.36	3.44	0.99
L3	17.62 ± 6.28	−16.73 ± 7.13	286.03 ± 79.83	−311.94 ± 90.01	−122.24 ± 10.89	10.06	0.99
L4	−75.18 ± 15.37	82.22 ± 21.56	−630.88 ± 240.36	825.48 ± 297.26	34.37 ± 14.68	42.23	0.99
L5	13.55 ± 5.24	−5.34 ± 3.29	970.40 ± 88.42	−958.31 ± 109.75	−657.22 ± 76.19	49.27	0.99
L6	−28.99 ± 11.04	44.15 ± 15.37	438.50 ± 83.15	−383.14 ± 94.26	−397.83 ± 76.26	90.95	0.99
M1	77.47 ± 19.48	−76.19 ± 25.49	1903.09 ± 406.49	−2102.02 ± 199.83	−758.29 ± 270.48	7.70	0.99
M2	−46.09 ± 4.27	49.51 ± 8.46	−549.87 ± 184.76	657.18 ± 199.86	131.80 ± 44.75	20.51	0.99
M3	42.94 ± 2.91	−38.24 ± 2.85	954.96 ± 215.20	−1003.28 ± 229.42	−528.57 ± 86.72	28.20	0.99
M4	−33.94 ± 2.04	47.25 ± 11.87	−552.73 ± 67.20	725.91 ± 88.44	−30.97 ± 3.05	79.84	0.99
M5	72.60 ± 17.47	−50.13 ± 7.68	2409.77 ± 315.59	−2534.83 ± 299.35	−1343.89 ± 164.78	134.79	0.99
M6	−58.93 ± 7.27	71.96 ± 14.89	−958.59 ± 119.28	1211.22 ± 186.47	60.52 ± 27.48	78.19	0.99
H1	107.34 ± 26.97	−106.95 ± 9.56	2613.89 ± 382.19	−2926.57 ± 351.00	−987.25 ± 97.45	2.35	0.99
H2	−6.54 ± 1.89	11.42 ± 2.78	551.03 ± 98.47	−536.62 ± 97.24	−372.27 ± 52.49	29.25	0.99
H3	−37.50 ± 9.35	48.38 ± 8.14	3.61 ± 1.70	98.32 ± 24.16	−226.09 ± 83.67	65.25	0.99
H4	43.11 ± 6.11	−40.48 ± 8.01	1437.75 ± 208.36	−1559.25 ± 216.30	−597.67 ± 92.65	15.78	0.99
H5	−113.29 ± 25.63	132.24 ± 31.27	−832.41 ± 122.47	1092.80 ± 189.48	−27.36 ± 9.46	113.73	0.99
H6	184.96 ± 36.18	−164.70 ± 25.33	1610.55 ± 44.02	−1855.61 ± 76.81	−449.15 ± 92.87	121.56	0.99

**Table 5:** Summary of dominant trends observed across formulation parameters.

Outcome	CaCl_2_ Concentration	Alginate Viscosity Grade	Alginate Concentration
Pore size	Decrease	Decrease	Slight decrease
Swelling	Reduced at higher crosslinking (evaluated at fixed CaCl_2_)	Increase	No significant effect
Water uptake	Decrease (at higher CaCl_2_)	Interaction-dependent	Decrease
Storage modulus (E′)	Increase	Increase	Increase
Hysteresis ratio (HR)	Increase	Increase	Increase
Cell viability	Decrease	Formulation-dependent	Viscosity-grade-dependent

**Table 6:** Different concentrations of alginate hydrogel formulations prepared for the experiments.

Number	Name	Polymer Viscosity	Polymer Concentration (*w*/*v*%)	Cross-Linker Concentration (*w*/*v*%)
1	L1	Low	1.0	2.5
2	L2			5.0
3	L3			10.0
4	L4		2.0	2.5
5	L5			5.0
6	L6			10.0
7	M1	Medium	1.0	2.5
8	M2			5.0
9	M3			10.0
10	M4		2.0	2.5
11	M5			5.0
12	M6			10.0
13	H1	High	0.5	2.5
14	H2			5.0
15	H3			10.0
16	H4		1.0	2.5
17	H5			5.0
18	H6			10.0

## Data Availability

The original contributions presented in this study are included in the article/Supplementary Materials. Further inquiries can be directed to the corresponding author.

## Supplementary Materials

The following supporting information can be downloaded at: https://www.mdpi.com/article/10.3390/ph19091441/s1, Table S1: ANOVA results for Pore size; Table S2: ANOVA results for water uptake; Table S3: ANOVA results for Storage Modulus; Table S4: ANOVA results for loss Modulus; Table S5: ANOVA results for Complex Modulus; Table S6: ANOVA results for Young’s modulus; Table S7: ANOVA results for Delta; Table S8: ANOVA results for Hysteresis Ratio; Table S9: ANOVA results for Live cells; Figure S1: Representative data from cyclic compression testing of alginate hydrogels; Figure S2: Additional representative scanning electron microscopy (SEM) micrographs of alginate hydrogels with different viscosity grades; Figure S3: Representative images supporting qualitative and quantitative assessment of cell viability after 20 days of culture.
