# Supplementary material for: Comparative Evaluation of Commercial Alginate Hydrogels: Effects of Viscosity, Polymer Concentration, and Crosslinking on Structural, Mechanical, and Biological Properties
Source: Pharmaceuticals (Basel). 2026 Sep 11;19(9):1441. doi: 10.3390/ph19091441 (PMC13610700; doi:10.3390/ph19091441)
Supplement: Supplementary file 1 [file pharmaceuticals-19-01441-s001.zip › pharmaceuticals-4481202-supplementary.pdf]

pharmaceuticals

MDPI

| Two-way ANOVA results for pore size | | | | | |
|---|---|---|---|---|---|
| Analysis | Source | df | F | p-value | Partial $\eta^2$ |
| 1% alginate | $CaCl_2$ concentration | 2 | 504.830 | <0.001 | 0.966 |
| | Alginate viscosity grade | 2 | 664.388 | <0.001 | 0.974 |
| | $CaCl_2$ × viscosity grade | 4 | 53.219 | <0.001 | 0.855 |
| Low viscosity | $CaCl_2$ concentration | 2 | 590.092 | <0.001 | 0.980 |
| | Alginate concentration | 1 | 147.577 | <0.001 | 0.860 |
| | $CaCl_2$ × alginate concentration | 2 | 24.850 | <0.001 | 0.674 |
| Medium viscosity | $CaCl_2$ concentration | 2 | 241.152 | <0.001 | 0.953 |
| | Alginate concentration | 1 | 310.851 | <0.001 | 0.928 |
| | $CaCl_2$ × alginate concentration | 2 | 3.535 | 0.045 | 0.228 |
| High viscosity | $CaCl_2$ concentration | 2 | 589.712 | <0.001 | 0.980 |
| | Alginate concentration | 1 | 114.250 | <0.001 | 0.826 |
| | $CaCl_2$ × alginate concentration | 2 | 22.889 | <0.001 | 0.656 |

*Table S1. ANOVA results for Pore size*

| Two-way ANOVA results for water uptake | | | | | |
|---|---|---|---|---|---|
| Analysis | Source | df | F | p-value | Partial $\eta^2$ |
| 1% alginate | $CaCl_2$ concentration | 2 | 8.856 | <0.001 | 0.330 |
| | Alginate viscosity grade | 2 | 12.965 | <0.001 | 0.419 |
| | $CaCl_2$ × viscosity grade | 4 | 1.901 | 0.132 | 0.174 |
| Low viscosity | $CaCl_2$ concentration | 2 | 0.850 | 0.440 | 0.066 |
| | Alginate concentration | 1 | 33.421 | <0.001 | 0.582 |
| | $CaCl_2$ × alginate concentration | 2 | 5.515 | 0.011 | 0.315 |
| Medium viscosity | $CaCl_2$ concentration | 2 | 2.939 | 0.072 | 0.197 |
| | Alginate concentration | 1 | 0.425 | 0.521 | 0.017 |
| | $CaCl_2$ × alginate concentration | 2 | 1.443 | 0.256 | 0.107 |
| High viscosity | $CaCl_2$ concentration | 2 | 7.197 | 0.004 | 0.375 |
| | Alginate concentration | 1 | 11.305 | 0.003 | 0.320 |
| | $CaCl_2$ × alginate concentration | 2 | 1.424 | 0.260 | 0.106 |

*Table S2. ANOVA results for water uptake*

| Two-way ANOVA results for Storage Modulus (E′) | | | | | |
|---|---|---|---|---|---|
| Analysis | Source | df | F | p-value | Partial $\eta^2$ |
| 1% alginate | $CaCl_2$ concentration | 2 | 26.487 | <0.001 | 0.595 |
| | Alginate viscosity grade | 2 | 184.695 | <0.001 | 0.911 |
| | $CaCl_2$ × viscosity grade | 4 | 15.657 | <0.001 | 0.635 |
| Low viscosity | $CaCl_2$ concentration | 2 | 11.943 | <0.001 | 0.499 |
| | Alginate concentration | 1 | 741.389 | <0.001 | 0.969 |
| | $CaCl_2$ × alginate concentration | 2 | 5.116 | 0.014 | 0.299 |
| Medium viscosity | $CaCl_2$ concentration | 2 | 58.353 | <0.001 | 0.829 |
| | Alginate concentration | 1 | 821.964 | <0.001 | 0.972 |
| | $CaCl_2$ × alginate concentration | 2 | 43.819 | <0.001 | 0.785 |
| High viscosity | $CaCl_2$ concentration | 2 | 50.585 | <0.001 | 0.808 |
| | Alginate concentration | 1 | 6.513 | 0.017 | 0.213 |
| | $CaCl_2$ × alginate concentration | 2 | 0.190 | 0.828 | 0.016 |

*Table S3. ANOVA results for Storage Modulus*

| Two-way ANOVA results for Loss Modulus (E″) | | | | | |
|---|---|---|---|---|---|
| Analysis | Source | df | F | p-value | Partial $\eta^2$ |
| 1% alginate | $CaCl_2$ concentration | 2 | 5.478 | 0.008 | 0.233 |
| | Alginate viscosity grade | 2 | 31.648 | <0.001 | 0.637 |
| | $CaCl_2$ × viscosity grade | 4 | 3.627 | 0.014 | 0.287 |
| Low viscosity | $CaCl_2$ concentration | 2 | 6.984 | 0.004 | 0.368 |
| | Alginate concentration | 1 | 80.838 | <0.001 | 0.771 |
| | $CaCl_2$ × alginate concentration | 2 | 6.023 | 0.008 | 0.334 |
| Medium viscosity | $CaCl_2$ concentration | 2 | 10.434 | <0.001 | 0.465 |
| | Alginate concentration | 1 | 136.320 | <0.001 | 0.850 |
| | $CaCl_2$ × alginate concentration | 2 | 6.129 | 0.007 | 0.338 |
| High viscosity | $CaCl_2$ concentration | 2 | 22.409 | <0.001 | 0.651 |
| | Alginate concentration | 1 | 0.022 | 0.883 | 0.001 |
| | $CaCl_2$ × alginate concentration | 2 | 1.430 | 0.259 | 0.106 |

*Table S4. ANOVA results for loss Modulus*

| Two-way ANOVA results for Complex Modulus (E*) | | | | | |
|---|---|---|---|---|---|
| Analysis | Source | df | F | p-value | Partial $\eta^2$ |
| 1% alginate | $CaCl_2$ concentration | 2 | 81.493 | <0.001 | 0.819 |
| | Alginate viscosity grade | 2 | 564.255 | <0.001 | 0.969 |
| | $CaCl_2$ × viscosity grade | 4 | 39.922 | <0.001 | 0.816 |
| Low viscosity | $CaCl_2$ concentration | 2 | 4.302 | 0.025 | 0.264 |
| | Alginate concentration | 1 | 197.789 | <0.001 | 0.892 |
| | $CaCl_2$ × alginate concentration | 2 | 2.205 | 0.132 | 0.155 |
| Medium viscosity | $CaCl_2$ concentration | 2 | 54.839 | <0.001 | 0.820 |
| | Alginate concentration | 1 | 674.334 | <0.001 | 0.966 |
| | $CaCl_2$ × alginate concentration | 2 | 25.378 | <0.001 | 0.679 |
| High viscosity | $CaCl_2$ concentration | 2 | 103.712 | <0.001 | 0.896 |
| | Alginate concentration | 1 | 11.952 | 0.002 | 0.332 |
| | $CaCl_2$ × alginate concentration | 2 | 0.547 | 0.586 | 0.044 |

*Table S5. ANOVA results for Complex Modulus*

| Two-way ANOVA results for Young's Modulus (YM) | | | | | |
|---|---|---|---|---|---|
| Analysis | Source | df | F | p-value | Partial $\eta^2$ |
| 1% alginate | $CaCl_2$ concentration | 2 | 476.818 | <0.001 | 0.964 |
| | Alginate viscosity grade | 2 | 4035.249 | <0.001 | 0.996 |
| | $CaCl_2$ × viscosity grade | 4 | 283.291 | <0.001 | 0.969 |
| Low viscosity | $CaCl_2$ concentration | 2 | 125.414 | <0.001 | 0.913 |
| | Alginate concentration | 1 | 7231.786 | <0.001 | 0.997 |
| | $CaCl_2$ × alginate concentration | 2 | 89.991 | <0.001 | 0.882 |
| Medium viscosity | $CaCl_2$ concentration | 2 | 215.770 | <0.001 | 0.947 |
| | Alginate concentration | 1 | 3809.439 | <0.001 | 0.994 |
| | $CaCl_2$ × alginate concentration | 2 | 158.001 | <0.001 | 0.929 |
| High viscosity | $CaCl_2$ concentration | 2 | 1121.793 | <0.001 | 0.989 |
| | Alginate concentration | 1 | 966.906 | <0.001 | 0.976 |
| | $CaCl_2$ × alginate concentration | 2 | 10.018 | <0.001 | 0.455 |

*Table S6. ANOVA results for Young's modulus*

| Two-way ANOVA results for Phase Angle (δ) | | | | | |
|---|---|---|---|---|---|
| Analysis | Source | df | F | p-value | Partial $\eta^2$ |
| 1% alginate | $CaCl_2$ concentration | 2 | 1.361 | 0.269 | 0.070 |
| | Alginate viscosity grade | 2 | 5.089 | 0.011 | 0.220 |
| | $CaCl_2$ × viscosity grade | 4 | 0.329 | 0.857 | 0.035 |
| Low viscosity | $CaCl_2$ concentration | 2 | 2.898 | 0.075 | 0.195 |
| | Alginate concentration | 1 | 0.227 | 0.638 | 0.009 |
| | $CaCl_2$ × alginate concentration | 2 | 6.490 | 0.006 | 0.351 |
| Medium viscosity | $CaCl_2$ concentration | 2 | 1.566 | 0.230 | 0.115 |
| | Alginate concentration | 1 | 2.055 | 0.165 | 0.079 |
| | $CaCl_2$ × alginate concentration | 2 | 1.939 | 0.166 | 0.139 |
| High viscosity | $CaCl_2$ concentration | 2 | 0.928 | 0.409 | 0.072 |
| | Alginate concentration | 1 | 5.847 | 0.024 | 0.196 |
| | $CaCl_2$ × alginate concentration | 2 | 1.646 | 0.214 | 0.121 |

*Table S7. ANOVA results for Delta*

| Two-way ANOVA results for Hysteresis Ratio (HR) | | | | | |
|---|---|---|---|---|---|
| Analysis | Source | df | F | p-value | Partial $\eta^2$ |
| 1% alginate | $CaCl_2$ concentration | 2 | 30.166 | <0.001 | 0.626 |
| | Alginate viscosity grade | 2 | 228.762 | <0.001 | 0.927 |
| | $CaCl_2$ × viscosity grade | 4 | 26.821 | <0.001 | 0.749 |
| Low viscosity | $CaCl_2$ concentration | 2 | 45.457 | <0.001 | 0.791 |
| | Alginate concentration | 1 | 81.683 | <0.001 | 0.773 |
| | $CaCl_2$ × alginate concentration | 2 | 36.219 | <0.001 | 0.751 |
| Medium viscosity | $CaCl_2$ concentration | 2 | 4.818 | 0.017 | 0.287 |
| | Alginate concentration | 1 | 75.397 | <0.001 | 0.759 |
| | $CaCl_2$ × alginate concentration | 2 | 8.763 | 0.001 | 0.422 |
| High viscosity | $CaCl_2$ concentration | 2 | 12.982 | <0.001 | 0.520 |
| | Alginate concentration | 1 | 115.604 | <0.001 | 0.828 |
| | $CaCl_2$ × alginate concentration | 2 | 17.911 | <0.001 | 0.599 |

*Table S8. ANOVA results for Hysteresis Ratio*

| Two-way ANOVA results for Live Cells | | | | | |
|---|---|---|---|---|---|
| Analysis | Source | df | F | p-value | Partial $\eta^2$ |
| 1% alginate | $CaCl_2$ concentration | 2 | 571.496 | <0.001 | 0.970 |
| | Alginate viscosity grade | 2 | 167.318 | <0.001 | 0.903 |
| | $CaCl_2$ × viscosity grade | 4 | 10.936 | <0.001 | 0.549 |
| Low viscosity | $CaCl_2$ concentration | 2 | 331.579 | <0.001 | 0.965 |
| | Alginate concentration | 1 | 157.932 | <0.001 | 0.868 |
| | $CaCl_2$ × alginate concentration | 2 | 15.749 | <0.001 | 0.568 |
| Medium viscosity | $CaCl_2$ concentration | 2 | 349.662 | <0.001 | 0.967 |
| | Alginate concentration | 1 | 121.826 | <0.001 | 0.835 |
| | $CaCl_2$ × alginate concentration | 2 | 8.388 | 0.002 | 0.411 |
| High viscosity | $CaCl_2$ concentration | 2 | 321.084 | <0.001 | 0.964 |
| | Alginate concentration | 1 | 61.743 | <0.001 | 0.720 |
| | $CaCl_2$ × alginate concentration | 2 | 5.632 | 0.010 | 0.319 |

*Table S9. ANOVA results for Live cells*

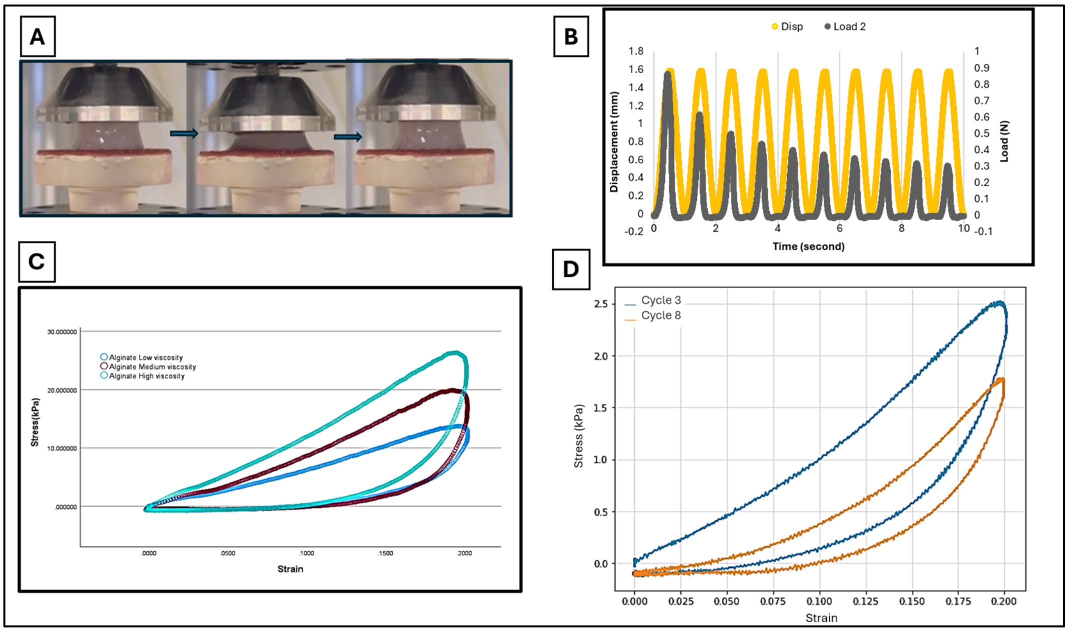


Figure S1. Representative data from cyclic compression testing of alginate hydrogels. (A) Sequential images of a hydrogel specimen during unconfined cyclic compression. (B) Displacement and load signals recorded during a 10-cycle compression test at 1 Hz. (C) Stress–strain hysteresis loops for low-, medium-, and high-viscosity alginate hydrogels, showing the effect of viscosity on viscoelastic response. (D) Stress–strain hysteresis loops from the third and eighth loading cycles of a hydrogel sample. The reduced hysteresis loop area between these cycles indicates a progressive decrease in energy dissipation due to cyclic mechanical preconditioning. Cycles 3 and 8 were chosen to illustrate changes in viscoelastic response during cyclic compression.

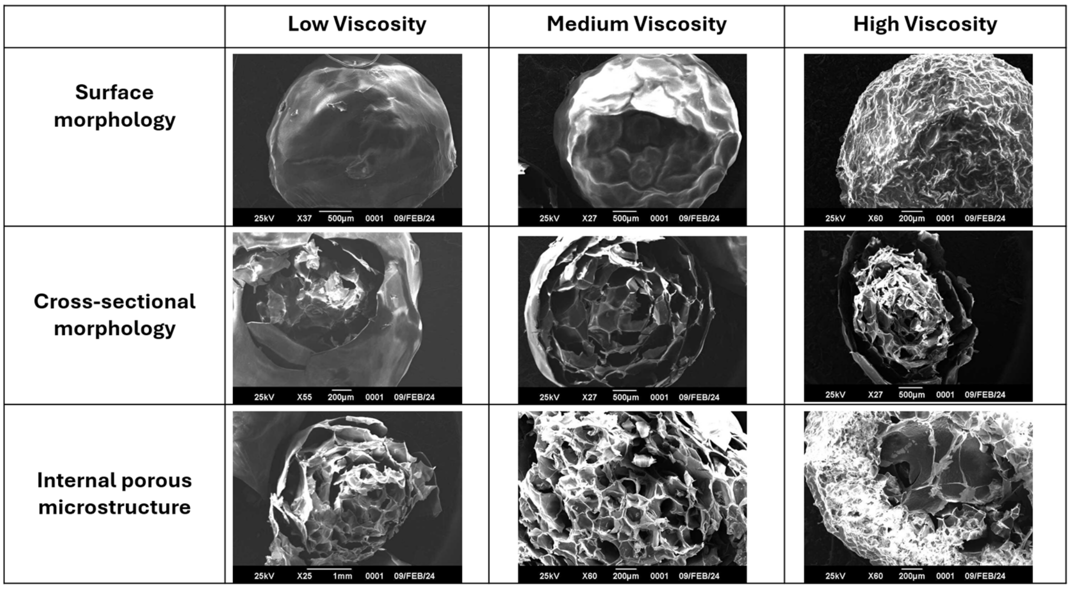


Figure S2. Additional representative scanning electron microscopy (SEM) micrographs of alginate hydrogels with different viscosity grades. Representative SEM images illustrating the surface morphology (top row), cross-sectional morphology (middle row), and internal pore morphology

(bottom row) of low-, medium-, and high-viscosity alginate hydrogels. These additional micrographs complement the representative SEM images presented in the main manuscript and provide further visualization of the structural heterogeneity and porous microstructure of the hydrogel network across different viscosity grades.

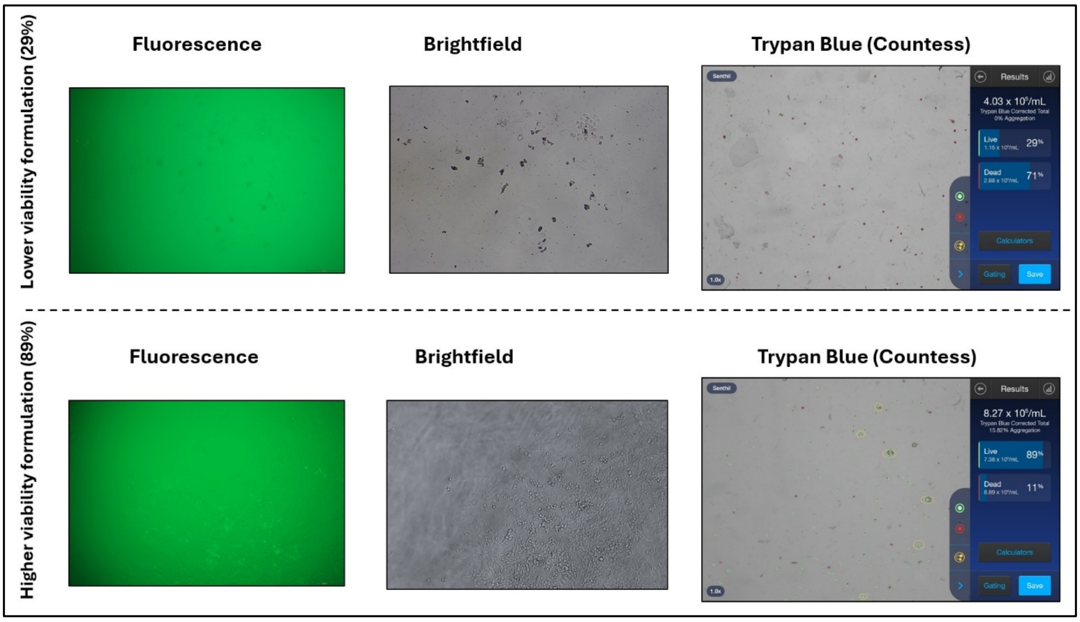


Figure S3. Representative images supporting qualitative and quantitative assessment of cell viability after 20 days of culture. Representative fluorescence images (left), corresponding bright-field images (middle), and trypan blue exclusion assay images obtained using an automated cell counter (right) are shown for hydrogel formulations showing lower (29%) and higher (89%) cell viability. GFP fluorescence was weak under the imaging conditions used. Therefore, fluorescence images are provided for qualitative visualization only, while quantitative cell viability was determined using the trypan blue exclusion assay. Representative images are consistent with the quantitative viability results reported in the original manuscript.
